# RNA Modifications: Current Understandings and Future Perspectives

**DOI:** 10.1002/mco2.70734

**Published:** 2026-04-19

**Authors:** Shiyu Xiao, Tingwen Xiang, Chuan Yang, Xiaohua Wang, Gang Huang, Fei Luo, Zhao Xie, Yueqi Chen

**Affiliations:** ^1^ Department of Orthopedics, Southwest Hospital Third Military Medical University (Army Medical University) Chongqing People's Republic of China; ^2^ Department of Biomedical Materials Science Third Military Medical University (Army Medical University) Chongqing People's Republic of China; ^3^ Department of Biochemistry and Molecular Biology, College of Basic Medical Science Third Military Medical University (Army Medical University) Chongqing People's Republic of China; ^4^ Senior Department of Orthopedics The Fourth Medical Center of Chinese PLA General Hospital Beijing People's Republic of China; ^5^ Department of Orthopedics Chinese PLA 76th Army Corps Hospital Xining People's Republic of China

**Keywords:** epigenetic modification, homeostasis, N4‐acetylcytidine, pathophysiology, RNA modification

## Abstract

RNA modification has been established as a pivotal field in epitranscriptomics, representing an emerging, dynamic, and precise regulatory layer in gene expression control. N6‐methyladenosine (m^6^A), the most prevalent internal RNA modification, is critical for post‐transcriptional regulation of RNA stability, translation, and degradation. In addition to m^6^A, RNA contains a number of other modifications that play important regulatory roles in RNA metabolism, transport, translation, and stability. Our review uses N4‐acetylcytidine (ac4C) modification as a research paradigm to conduct a systematic review of the RNA modification research framework. This article begins with RNA modifications, then discusses several RNA modification‐related regulatory enzymes before using ac4C as a detailed research example. Starting with the fundamental functions of ac4C in RNA modifications, it discusses its discovery history, the specific mechanisms of the key acetyltransferase N‐acetyltransferase 10 (NAT10) in various RNA modifications, existing detection technologies, and the functional significance of ac4C modification under physiological and pathological conditions. This review systematically explains the multidimensional roles of RNA modifications, represented by ac4C, in health and disease. We point out that RNA modification‐related regulatory enzymes, such as NAT10, can serve as prognostic biomarkers and therapeutic targets, thereby advancing disease mechanism research and improving clinical diagnosis and treatment.

## Graphical abstract

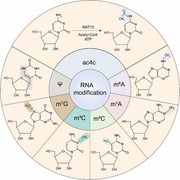

## 1 Introduction

The process of gene expression is the foundation for cellular function and the development of organisms. The complexity of regulatory mechanisms for gene expression is as great as the variety of biological processes. Epigenetics is one of the many regulatory mechanisms of inheritable change in gene expression or cellular phenotype that occurs without a change in the DNA sequence. Simultaneously, post‐transcriptional gene regulation (PTGR) is another major player in the flow of genetic information, affecting RNA maturation, transport, stability, and translation of both coding and noncoding RNAs. This is widely recognized as being integral to disease mechanisms. RNA‐binding proteins and ribonucleoproteins play an important coordinating role in RNA processing and PTGR [1, 2]. To date, over 170 types of PTGR mechanisms have been identified, primarily grouped into two categories: those affecting ribosomal RNA (rRNA) and those affecting transfer RNA (tRNA). Among these, the rRNA modifications are most abundant, while the tRNA modifications show maximum diversity [3].

RNA modifications encompass a diverse array of covalent chemical alterations to nucleotide bases across various RNA species, serving as fundamental regulators of gene expression [4]. Owing to their widespread prevalence and functional significance, specific modifications including m^6^A, 5‐methylcytidine (m^5^C), N1‐methyladenosine (m^1^A), and ac4C have garnered considerable scientific interest. These chemical marks act as crucial regulatory determinants, influencing RNA stability, transcriptional output, and translational efficiency, thereby governing a multitude of cellular and physiological processes [5, 6]. For example, m^6^A modification demonstrates effects on decoding dynamics during protein synthesis [7, 8]. Furthermore, m^6^A deposition exhibits remarkable plasticity in response to external signals, facilitating rapid adaptation of cellular gene expression programs to environmental perturbations [8, 9]. Another significant modification, pseudouridine (ψ), is dynamically incorporated into human mRNAs and is instrumental in mRNA processing and alternative splicing. Accumulating data indicate that ψ exerts regulatory influence at multiple stages of the pre‐mRNA lifecycle; it can, for instance, directly modulate splicing efficiency to precisely adjust gene expression patterns [10]. This highlights the context‐dependent nature of RNA modification functions, which are shaped by specific cellular environments and molecular interaction networks [9]. Consequently, the expression profiles and functional impacts of m^6^A and other modifications can vary substantially across different tissues or cell types. These modification signatures are frequently decoded in a cell‐specific manner, resulting in distinct downstream biological consequences [11]. In summary, RNA modifications constitute an evolutionarily conserved layer of chemical information whose functional diversity stems from precise spatiotemporal deployment within RNA metabolic pathways.

Ac4C represents an emerging and increasingly characterized acetylation mark within the epitranscriptomic landscape [12]. This modification exhibits a broad distribution across diverse RNA species, with its predominant enrichment localized within the 5′ coding sequences and less frequently observed in 3′ untranslated regions (3′ UTRs) [13]. Initially identified in tRNA, ac4C has been subsequently mechanistically linked to its primary writer enzyme, NAT10 [14, 15, 16]. The seminal investigation by Arango et al., which delineated ac4C in mRNA, constituted a pivotal breakthrough, catalyzing a substantial influx of studies that associate this modification with a wide spectrum of human pathologies [17]. Contemporary research has progressively elucidated the role of ac4C modifications in biosynthetic pathways, fundamental processes underpinning cellular homeostasis [18, 19]. Systems governed by this modification are recognized to modulate RNA stability and translational efficiency, leveraging the distinctive physicochemical properties conferred by acetylation. These alterations are postulated to facilitate enhanced protein synthesis and metabolic flux. Ultimately, by augmenting molecular resilience, ac4C‐mediated changes contribute to extended RNA persistence and functional efficacy within the cellular milieu [20, 21].

This review systematically synthesizes current knowledge on RNA modification mechanisms, employing ac4C as a central exemplar. It delineates the establishment and functional impacts of RNA modifications across diverse physiological conditions and pathological states in biological systems. Progress in targeting epitranscriptomic marks and their regulatory enzymes is paving the way for promising next‐generation therapeutic strategies in human diseases.

## 2 The Diversity and Detection of RNA Modifications

RNA modifications encompass a diverse array of chemical alterations that occur on ribonucleic acid molecules. These modifications exert a pivotal regulatory influence on gene expression by modulating RNA structural dynamics, metabolic stability, translational fidelity, and intermolecular interactions within the cellular milieu. The establishment, interpretation, and removal of these marks are catalyzed by specialized enzymes—writers, erasers, and readers, respectively. These actors operate in a coordinated manner to constitute a sophisticated and dynamic epitranscriptomic regulatory network.

### 2.1 A Landscape of Major RNA Modifications and Their Writers, Erasers, Readers

**FIGURE 1:**
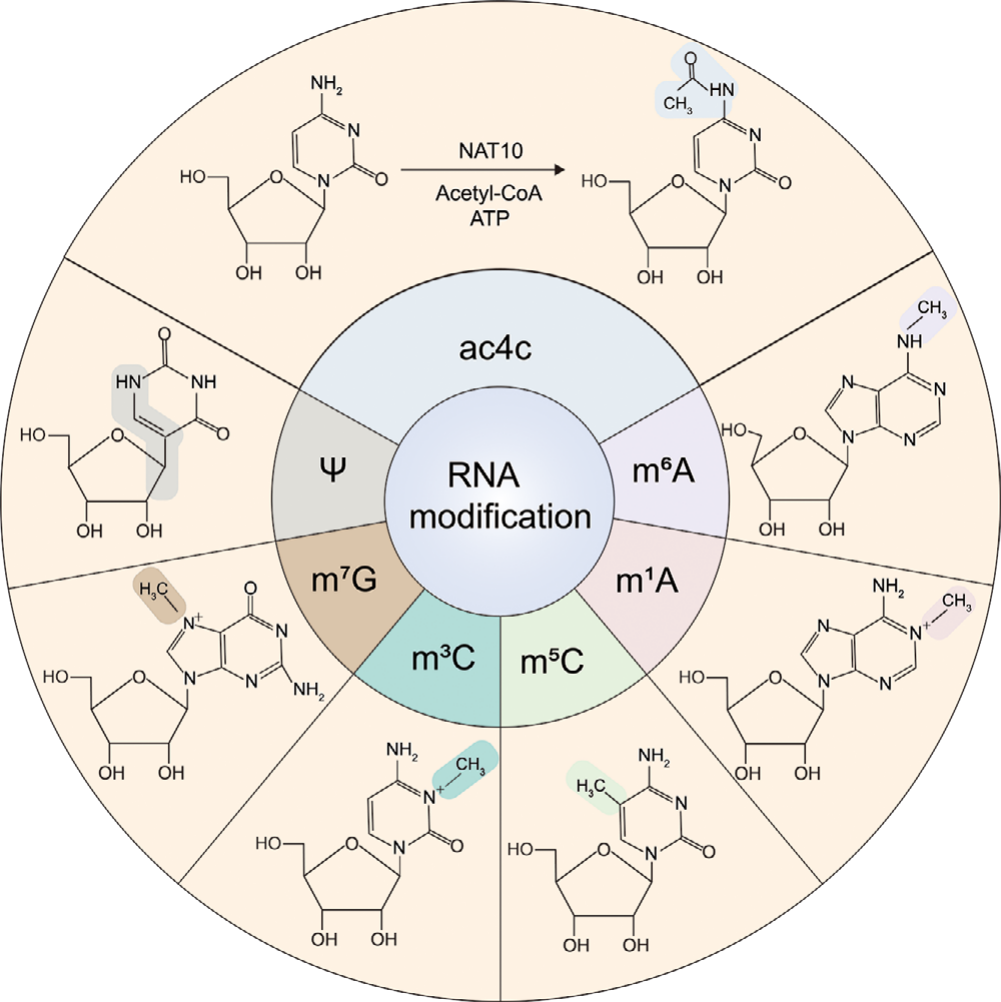
Types of RNA modification. We have summarized the currently common types of RNA modifications, including ac4C, m^6^A, m^1^A, m^5^C, m^3^C, m^7^G, and ψ, and visually characterized their features through structural formulas. The characteristic structures are marked with a background color different from the background color.

In RNA modification, writers, erasers, and readers are three classes of proteins that work together to regulate the chemical modifications of RNA (Figure 1). The activities of these three classes of proteins, which influence RNA metabolism and function, underscore the regulation of gene expression, cell fate determination, and the onset of pathology.

Writers are enzymes that can add specific chemical modifications to RNA molecules. The main writers of m^6^A modification are the m^6^A methyltransferase complex, including METTL3, METTL14, WTAP, RBM15/15B, VIRMA, and ZC3H13. These enzymes methylate RNA at specific sites to produce m^6^A modifications [17]. The task of writers is not simply the addition of methyl groups to RNA but also the selective recognition of RNA structure and sequence to influence which RNA gets modified. The selectivity is also dependent on the cell types and the environment in which it occurs [9]. For instance, the expression levels and patterns of m^6^A can vary among various cell types to influence their RNA stability, translation efficiency, and degradation rates [9, 22].

RNA modification is a dynamic process. It is mediated through enzymes known as “erasers,” which catalyze the removal of certain chemical groups from nucleotides. We will use m^6^A as an example to explain the characteristic. The fat mass and obesity‐associated protein (FTO) and AlkB homolog 5 (ALKBH5) are the main demethylases that remove m^6^A methylation. By carrying out oxidative demethylation, these enzymes alter the chemical structure of the modified RNA and thus influence its biological activity [17, 23]. These erasers exert a drastic effect on RNA metabolism, which works mainly by altering transcript stability and translational efficiency. According to experimental evidence, the removal of m^6^A modifications may change RNA fate, for instance by promoting mRNA decay and inhibiting protein synthesis. This phenomenon is important for the coordination of adaptation mechanisms of cells to environmental stress response as well as for RNA life cycle regulation [23, 24]. Dysfunction of RNA modification erasers is implicated in several diseases. FTO is found to be overexpressed in multiple cancer types, meaning its overabundance leads to excessive removal of m^6^A marks from the transcripts of tumor suppressor genes, suppressing protective genes. When this takes place, it gives tumor cells a selective edge that allows them to proliferate more and undergo programmed cell death less [23].

In RNA, readers can recognize and bind specific changes. Under normal circumstances, these proteins allow mediation of downstream actions of proteins. For example, several major readers—YTHDC1, YTHDF1/2/3, IGF2BP1/2/3, and HNRNPA2B1—are involved in m^6^A modification. The fate of RNAs is modulated through interaction and binding to m^6^A. It includes post‐transcriptional modifications, translation, localization, degradation, and so on [17]. Enhanced RNA triggers differential physiological responses in the readers. Some YTHDF proteins assist in the translation of m^6^A‐methylated RNA, while other readers may assist in their degradation. This complex interaction suggests RNA function is not predetermined but instead adjusts according to cell needs and external cues [22]. This reveals that m^6^A modification is flexible and actively involved in influencing cell response and decision‐making.

While m^6^A possesses a complete “write–read–erase” system, the regulation of ac4C remains far more enigmatic. The only known writer is the NAT10 acetyltransferase. Unlike m^6^A, the specific eraser for ac4C has not yet been identified in mRNA, suggesting that ac4C regulation may rely more on the expression activity of NAT10 or its deacetylation mechanism is highly unique. Additionally, the direct reader protein for ac4C remains a critical gap in the field, which limits our understanding of the downstream functional mechanisms.

Here, we list the common RNA modification writers, erasers, and readers (Table 1).

**TABLE 1:** Summary of RNA modification‐related enzymes.

RNA modification categories	Writers	Erasers	Readers	References
m^6^A	METTL3, METTL14, METTL16, WTAP, KIAA1429, RBM15, RBM15B, VIRMA, and ZC3H13	FTO, ALKBH3 and ALKBH5	YTHDF1, YTHDF2, YTHDF3, YTHDC1, YTHDC2, IGF2BP1/2/3, HNRNPC/G, HNRNPA2B1, and eIF3	[17, 25, 26, 27]
m^5^C	DNMT2, NSUN1, and NSUN2	/	TRDMT1 and YBX1	[25, 28]
m^1^A	TRMT6 and TRMT61A	/	IGF2BP family	[25, 28]
ac4C	NAT10	SIRT7	/	[16, 29]
ψ	PUS1, PUS7, and PUS10	/	/	[30, 31]

RNA modifications are governed by a dynamic system of enzymatic “writers,” “erasers,” and “readers.” Targeting distinct writer or eraser enzymes presents a strategy to modify disease manifestations and improve therapeutic outcomes [23, 24].

Although modifications often exhibit synergistic or competitive interactions, they each have distinct functional emphases. M^6^A is a versatile modifier that influences pre‐mRNA splicing, degradation, and translation [26, 32]. Ψ is primarily enriched in rRNA and tRNA, maintaining the precision and stability of the translation machinery and serving as a signaling molecule in innate immunity [31, 33]. Notably, m^5^C is not only an RNA modification but also a critical epigenetic modification in DNA, playing a pivotal role in regulating gene expression and disease progression [32, 34]. The known function of ac4C is highly specialized: on mRNA, it primarily enhances translation efficiency, particularly by improving codon preference to accelerate translation elongation. This specific regulation of translation elongation is a hallmark feature that distinguishes ac4C from other modifications such as m^6^A.

In summary, compared to other RNA modifications, ac4C exhibits a simplified regulatory mechanism and a specific functional bias. However, due to the limitations of detection techniques and the unknown nature of the reader protein, the regulatory network of ac4C remains unclear. This highlights the necessity for systematic research on ac4C.

### 2.2 Advanced Technologies for N4‐Acetylcytidine Detection

New methods for monitoring RNA modifications are pushing epitranscriptomics further ahead. The field of biological and bioinformatics research, which is critical, is the prediction of RNA modification sites. The detection technology for m^6^A has reached a high level of maturity, evolving from basic MeRIP‐seq to high‐resolution techniques like miCLIP, enabling single‐base localization. We will take ac4C modification as a typical example to introduce detection methods and prediction techniques in detail. Because ac4C modification can significantly affect RNA function, it is important to develop accurate detection methods to help locate and quantify ac4C. Strategies for detection have emerged rapidly due to this demand. In the past, the identification of ac4C was done mainly by experimental methods.

#### 2.2.1 Technological Detection Methods

The sodium borohydride cutback method targets nonhomologous dNTPs during reverse transcription and sequencing. However, it could not identify ac4C sites in RNA, such as tRNA, where codon changes had occurred [35, 36, 37].

Liquid chromatography (LC) initially served as the principal methodological framework for sequencing and characterizing ac4C modifications in RNA [38]. This approach is capable of delivering precise identification and quantitative evaluation of ac4C, dependent on differential analyte retention behavior during chromatographic separation [39]. Nevertheless, a major drawback lies in their inability to resolve site‐specific localization of RNA modifications at nucleotide resolution.

Mass spectrometry (MS) is another useful analytical technique in RNA research. MS facilitates precise measurements of mass‐to‐charge ratios of ions and provides crucial information concerning the molecular architecture of RNA [40]. Nevertheless, MS requires complex preparation, making it time‐consuming and degrading the sample.

The invention of acetylated RNA immunoprecipitation and sequencing (acRIP‐seq) technology made significant progress in RNA modification technology. This method starts with the fragmentation of RNA from cells or tissues, followed by incubation with anti‐ac4C. After several washes, the library construction and high‐throughput sequencing were performed on RNA fragments that bound specifically to the ac4C antibody [41]. The effectiveness of acRIP‐seq highly relies on the specificity and affinity of the antibody employed. The antibody's possible cross‐reactivity with unrelated but structurally similar modifications may give rise to false‐positive findings. Besides, it cannot pinpoint the ac4C modification site at the mononucleotide level accurately.

Ac4C‐specific sequencing (ac4C‐seq) enables the precise mapping of ac4C modifications at single‐nucleotide resolution [42]. The core mechanism utilizes an aminooxy‐derived oxime (ANTH) reagent, whose oxime group selectively reacts with the acetyl functional group of ac4C. The interaction facilitates site‐specific covalent tagging exclusively at ac4C residues. This design confers superior specificity, minimizing cross‐reactivity and false‐positive signals commonly associated with immunoprecipitation‐based techniques.

A new methodology named fluorine metabolic labeling‐mediated proximity ligation assay (FMPLA) could specifically detect ac4C modifications in cells. This novel strategy integrates metabolic incorporation of fluorine‐labeled precursors with in situ proximity ligation, allowing visualization and quantification of ac4C‐modified RNA [43]. However, the major caveat is that there is no direct comparative analysis of specificity with the already established “chemical mapping” ac4C‐seq (Table 2).

**TABLE 2:** Detection methods for ac4C modifications.

Methods	Description	Advantages	Limitations	References
Sodium borohydride reduction method	Detecting ac4C mark.	/	Cannot identify ac4C sites in RNA.	[35, 36, 37]
Liquid chromatography (LC)	The first principal method for sequencing RNA ac4C modifications.	Enable detailed identification and quantification of ac4C modification.	Unable to distinguish specific RNA modification sites.	[39]
High‐performance liquid chromatography (HPLC)	Changes in ac4C modification can be observed in disease models such as tumors.	Quantitative analysis of highly efficient ac4C modifications.	Unable to distinguish specific RNA modification sites.	[44, 45, 46]
Mass spectrometry (MS)	It can accurately measure the mass‐to‐charge ratio of ions, helping to gain deep insights into the molecular architecture of RNA.	1. High sensitivity and specificity. 2. Effective for quantitative analysis.	1. Require complex sample preparation, which can be time‐consuming and prone to sample loss or degradation. 2. Demand high‐quality equipment and significant expertise.	[40]
Liquid chromatography tandem‐mass spectrometry (LC–MS/MS)	Dual‐phase system: liquid chromatography separates compounds based on chemical properties, followed by tandem mass spectrometry's detailed analysis of molecular structures and masses.	1. Provide extensive structural information. 2. Can perform precise identification and quantification of complex biomolecules.	Demand a high level of expertise to operate the sophisticated equipment and interpret the complex data accurately.	[47]
High‐performance liquid chromatography mass Spectrometry (HPLC–MS)	First detection of ac4C modifications in 5S rRNA from thermophilic bacteria.	Great sensitivity and specificity to analyze a wide range of complex biological samples.	Operating the equipment is complex, and analyzing the data is difficult.	[48]
Partially enzymatic hydrolysis and two‐dimensional paper chromatography	Exploits the unique electronic structure of ac4C, which renders its 5,6‐double bond susceptible to nucleophilic attack by sodium borohydride.	1. Very sensitive and can be used to study the effectiveness of RNA acetylation stoichiometry. 2. Suitable for analysis ac4C sites of known rRNA and preribosomal RNA.	Lack of the ability to interrogate ac4C in more densely‐functionalized modification landscapes.	[49]
Acetylated RNA immunoprecipitation and sequencing	Genome‐wide detection of ac4C modifications can be accomplished in mammalian cells.	1. High‐throughput detection of the ac4C modification on cellular transcripts. 2. Modifications can be associated with a functional outcome.	Unable to accurately locate the ac4C modification site of the mononucleotide.	[41]
Ac4C‐seq	The first precise mapping of the ac4C modifications at the mononucleotide level.	High‐throughput detection of ac4C modifications at single‐nucleotide resolution.	1. The steps are complicated and difficult to operate. 2. The sensitivity is low and it has been suggested that ac4C modification on mRNA cannot be detected in mammals.	[42, 50]
RedaC: T‐seq	Map ac4C at base resolution through NaBH4‐induced ac4C reduction and conversion to thymidine, combined with sequencing.	The total ratio of ac4C to C in the transcriptome can be estimated by comparing the sequencing depth of the total C > T mismatch with that of reference cytidine.	Sodium borohydride can react with other bases. To call the ac4C sites, NAT10^−/−^ samples must be used.	[51, 52]
FAM‐seq	Fluorine‐assisted metabolic sequencing can identify the ac4C modification without antibody.	Antibody‐free methods to identify ac4C modification.	1. The prometabolite used in this method is highly toxic and lethal to most mammals and birds, making it difficult to conduct in vivo experiments. 2. Unable to accurately locate the ac4C modification site of the mononucleotide.	[53]
FMPLA	This method integrates metabolic fluorine labeling with proximity ligation to specifically label ac4C modifications.	Enables reliable in situ labeling of ac4C modifications.	No explicit detection accuracy data are available.	[43]

Nevertheless, these experimental approaches entail considerable technical complexity and demand substantial resources in terms of reagents, materials, and funding. Given the substantial progress in computational methodologies, the development of reliable computational prediction tools has emerged as an increasingly critical endeavor.

#### 2.2.2 Computational Prediction Tools

Advances in technology and computing have led researchers to combine computers and AI with experimental methods to improve the accuracy of predicting ac4C sites in mRNA. The field has evolved from early machine learning models (PACES with random forests, XG‐ac4C with XGBoost) to sophisticated deep learning approaches [54, 55]. CNN‐based models (DeepAc4C and CNNLSTMac4CPred) integrated physicochemical features, while hybrid architectures (EMDL‐ac4C, DLC‐ac4C, TransC‐ac4C, LSA‐ac4C, and DPNN‐ac4C) combined CNNs, LSTMs, and attention mechanisms for enhanced feature extraction [56, 57, 58, 59, 60, 61, 62]. Recent innovations employ transformers (TransAC4C and Voting‐ac4C), GANs (GANSamples‐ac4C and MetaAc4C) for data augmentation, extracting from RNA (iRNA‐ac4C and ac4C‐AFL), and pretrained language models (NBCR‐ac4C) for contextual embeddings [63, 64, 65, 66, 67, 68, 69]. Caps‐ac4C achieved state‐of‐the‐art accuracy by converting RNA sequences into visual representations processed by capsule networks [70]. Despite progress, challenges remain, including computational costs (ERNIE‐ac4C), limited generalizability to non‐mRNA RNAs, and data imbalance [71]. Current trends emphasize ensemble learning (Stacking‐ac4C), multihead attention (STM‐ac4C), and nanopore sequencing integration (ModCnet) for improved precision and scalability [70, 72, 73].

The accuracy and dependability of RNA modification detection models have improved, starting with PACES and moving on to Caps‐ac4C. As technologies advance in the future, more methods for detecting modifications such as ac4C will be widely accepted and could be integrated into sophisticated big data models driven by AI (Table 3).

**TABLE 3:** The prediction method of ac4C.

Methods	Description	References
PACES	PACES utilizes position‐specific dinucleotide sequence Spectra and K‐nucleotide frequencies as coding methods, with random forest deployed as a training model to yield the outcomes.	[54]
XG‑ac4C	The XG‐ac4C integrated model predicts ac4C locations using the eXtreme Gradient Boost classifier, utilizing electron–ion interaction pseudopotentials for individual nucleotides and trinucleotides at ac4C sites, along with Shapley additive and local interpretable model‐agnostic explanations to clarify feature importance and predictive impact.	[55]
DeepAc4C	DeepAc4C is built based on a convolutional neural network and a hybrid feature that integrated physicochemical patterns and nucleic acid distribution.	[56]
CNNLSTMac4CPred	Analyzed by using a mixture signature consisting of physicochemical patterns and nucleic acid distribution characterization.	[57]
EMDL‐ac4C	Used DenseNet in combination with convolutional residuals to form two‐branch residual connection DenseNet, and improve the effectiveness of feature extraction.	[74]
DLC‐ac4C	DLC‐ac4C is a prediction model for N4‐acetylcytidine sites in human mRNA utilizing DenseNet and bidirectional LSTM techniques.	[59]
LSA‐ac4C	LSA‐ac4C is a hybrid neural network incorporating double‐layer LSTM and self‐attention mechanism for the prediction of N4‐acetylcytidine sites.	[61]
Stacking‐ac4C	Stacking‐ac4C is a stacking‐based heterogeneous integrated ac4C model, which integrated three distinct feature extraction methodologies (Kmer, PseKNC, and PseEIIP) and a robust Cluster Centroids algorithm to improve the model adaptability.	[70]
iRNA‐ac4C	iRNA‐ac4C is a novel predictor based on three feature extraction methods, including nucleotide composition, nucleotide chemical property, and accumulated nucleotide frequency to identify ac4C sites in human mRNA.	[68]
NBCR‐ac4C	NBCR‐ac4C is a deep learning framework which was based on multivariate BERT.	[66]
GANSamples‐ac4C	The GANSamples‐ac4C framework represents a novel integration of transfer learning and Generative Adversarial Networks in the medical field.	[64]
MetaAc4C	MetaAc4C is a predictive model that leverages a bidirectional encoder (BERT) pretrained on transformers and Generative Adversarial Networks.	[65]
TransC‐ac4C	TransC‐ac4C integrates CNN and Transformer, employing five distinct feature encoding strategies to generate mRNA sequences.	[60]
TransAC4C	TransAC4C represents a pioneering interpretable framework for the multispecies identification of N4‐acetylcytidine sites in RNA, offering single‐base resolution.	[63]
Ac4C‐AFL	Ac4C‐AFL can precisely identify ac4C sites from primary RNA sequences.	[69]
STM‐ac4C	The STM‐ac4C model, based on selective kernel convolution, temporal convolutional networks, and multihead self‐attention, enhances its effectiveness in handling complex biological sequence data.	[72]
DPNN‐ac4C	DPNN‐ac4C is a Dual‐Path neural network with self‐attention mechanism.	[62]
Voting‐ac4C	Voting‐ac4C uses a pretrained large RNA language model to enhance RNA ac4C site prediction.	[67]
ModCnet	ModCnet could simultaneously and accurately detect ac4C and m^5^C modifications on mRNA.	[73]
ERNIE‐ac4C	ERNIE‐ac4C combined the ERNIE‐RNA language model and a two‐dimensional Convolutional CNN.	[71]
Caps‐ac4C	Caps‐ac4C utilizes chaos game representation to encode RNA sequences into “images” and employs capsule networks to learn global and local features from these RNA “images,” which is the most accurate tool for predicting ac4C sites in human mRNA.	[75]

Precise identification of RNA modifications and their locations is as critical as sequence determination, as these marks influence stability, localization, and translation. To manage complex epitranscriptomic data, researchers use AI‐driven computational models to predict modification sites, infer functions, and enhance discovery platforms, deepening mechanistic insights and accelerating biological discoveries.

## 3 Ac4C: A Paradigmatic Example for RNA Modifications

Ac4C, as a recently popular RNA modification research paradigm, shares many similarities with other RNA modifications. Its discovery process was also winding and full of exploratory significance. Different from the relatively comprehensive research on enzymes related to m^6^A modification, only NAT10 has been identified as the sole writer of ac4C, while SIRT7 can function as an eraser on non‐mRNA. No other enzymes have been identified yet, which awaits further research and exploration.

### 3.1 The Discovery and Conservation of Ac4C

Ac4C is an acetylation modification that occurs at the N4 position of the cytosine base within RNA molecules, acting as a conserved chemical alteration across a variety of RNA classes [14]. The ac4C modification has been shown to enhance the stability of modified mRNAs, decrease the rate of degradation, improve translation efficiency, and foster the expression of target genes [13, 20, 76]. Furthermore, it has the potential to enhance the accuracy of protein synthesis involving tRNAs and rRNAs.

The ac4C modification was first identified in 1966, following the pioneering structural analysis of two principal serine‐specific tRNAs from brewer's yeast [77]. Subsequent studies established its conservation across evolutionarily diverse organisms [78, 79, 80, 81, 82]. Nevertheless, the molecular mechanisms governing the establishment, removal, and functional regulation of ac4C modifications remain predominantly unresolved.

In 2004, Johansson demonstrated that TAN1 was essential for ac4C formation in tRNA. The TAN1 protein promoted ac4C incorporation specifically in tRNA via direct tRNA interaction, thereby contributing to tRNA^Ser^ stability through this modification. Although yeast TAN1 and its human homolog, TARBP1, share a conserved tRNA‐binding THUMP domain, TAN1 lacks a recognizable catalytic domain required for ac4C synthesis [81]. By 2008, the *Methanothermobacter thermautotrophicus* ortholog of *S. cerevisiae* TAN1, designated MTH909, was characterized as containing an N‐terminal ferredoxin‐like domain and a C‐terminal THUMP domain, yet it similarly lacked catalytic motifs [83]. These absences implied the involvement of an unidentified catalytic partner to complete the acetylation reaction. Concurrently, a genome‐wide screen in *E. coli* identified the tRNA^Met^ cytidine acetyltransferase (tmcA) [84]. TmcA was found to enable specific acetylation of the wobble cytidine in *E. coli* elongator tRNA^Met^ via consumption of acetyl‐CoA and ATP (or GTP) [84]. Interestingly, tmcA homologs were widely distributed among archaea and eukaryotes, and ac4C was found at the wobble positions of archaeal tRNAs and at position 12 in a subset of eukaryotic tRNAs. Based on these observations, the group of Tsutomu Suzuki hypothesized that the eukaryotic homolog of TmcA was an RNA acetyltransferase mediating ac4C formation in tRNA and/or 18S rRNA. Furthermore, in 2014, KRE33 was discovered and subsequently renamed as rRNA cytidine acetyltransferase 1 (RRA1), which encodes an RNA acetyltransferase that catalyzes the formation of ac4C at position 1773, utilizing ATP and acetyl‐CoA as substrates [44]. In a comparable manner, they reported in the same year that NAT10, a human homolog of Rra1p, functions as an ATP‐dependent RNA acetyltransferase accountable for ac4C formation at position 1842 in 18S rRNA of human HEK293 cells [44]. Since then, researchers have focused on the study of ac4C and NAT10 in human‐derived cells and diseases.

Sunny Sharma has certified that Kre33 and NAT10 are the tRNA acyltransferases in yeast and human cells, which are both required for small ribosomal subunit biogenesis. The yeast Kre33 and its human ortholog NAT10 exhibit a conserved modular architecture, characterized by a central domain of unknown function (DUF1726) flanked by an N‐terminal helicase domain (RecD), an acetyl‐CoA binding N‐acetyltransferase domain, and a C‐terminal tRNA‐binding motif. Structural and functional analyses suggest that the RecD helicase domain may function as a regulatory “molecular clamp,” modulating temporal access of substrate cytosines to the catalytic core [16]. Daniel Arango et al. proposed that ac4C represents the sole known RNA acetylation event in eukaryotes, with NAT10 serving as the unique human enzyme combining both acetyltransferase activity and RNA‐binding capacity. Their work demonstrated that ac4C is broadly distributed across the human transcriptome, predominantly enriched within coding sequences (CDS). Acetylation at these sites augments gene expression by enhancing mRNA stability and translational efficiency [13]. Notably, biophysical studies reveal that ac4C, when canonically paired with guanosine, confers increased thermal stability compared to unmodified cytosine. This thermodynamic advantage substantially augments the translational output of acetylated reporter mRNAs, an effect that is particularly pronounced when ac4C is incorporated at wobble positions within the codon–anticodon interface [13, 85].

**FIGURE 2:**
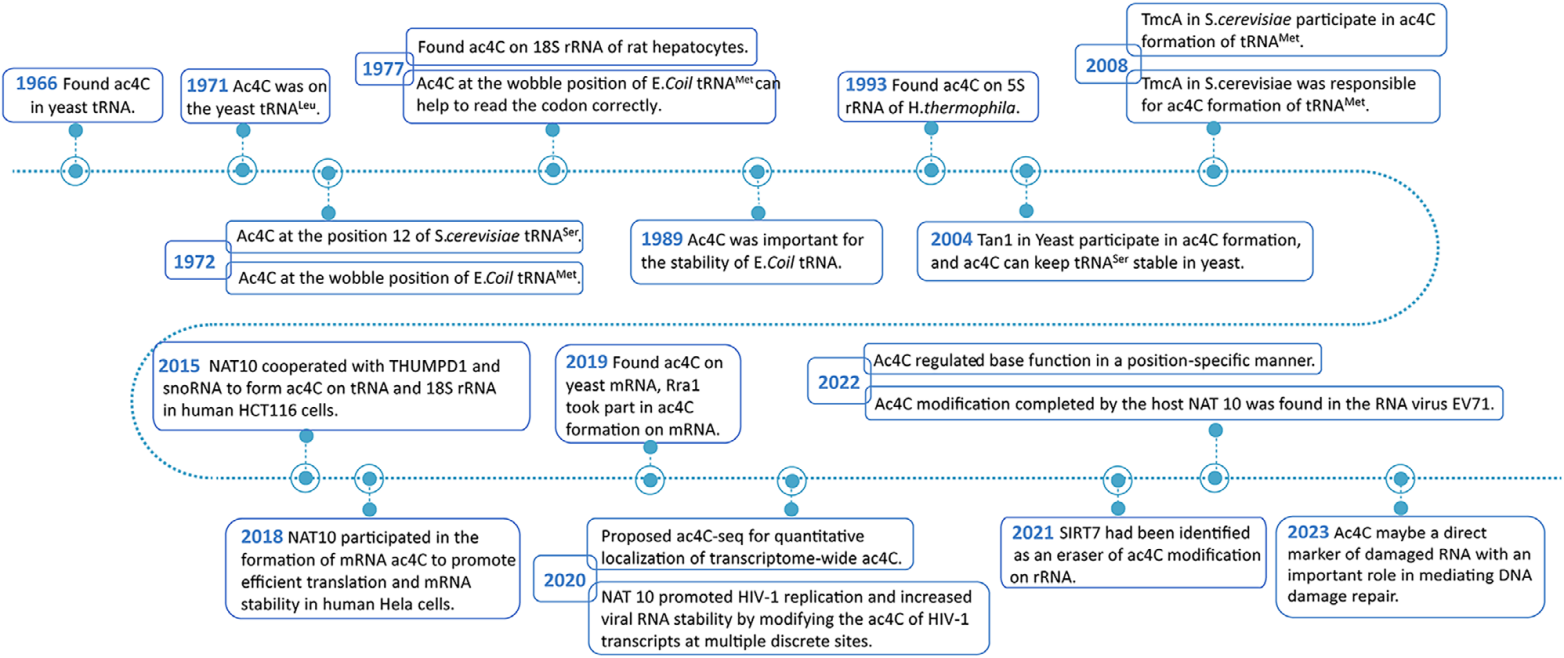
The history of ac4C and NAT10. The timeline encapsulates pivotal advances and the chronological sequence of breakthroughs in ac4C research. The timeline highlights key discoveries in ac4C research, from its initial identification in tRNA (1966) to its subsequent detection in rRNA and mRNA. It also marks the identification of key regulators, including the writer NAT10 and the eraser SIRT7, and the evolving understanding of ac4C's functions in RNA stability, translation, and DNA damage repair.

Ac4C modification plays an important role in different fields. In 1978, in vitro experiments using *E. coli* tRNA^Met^ suggested that ac4C at the wobble position prevents misreading of AUA isoleucine codons during protein synthesis [86]. In 1989, it was further determined that ac4C at the wobble position of tRNA^Met^ helped with the correct reading of the codon by stabilizing the C3′ ribose conformation in *E. coli* [87]. In 2004, it was discovered that the yeast *TAN1* gene was involved in ac4C formation on the tRNA and found that ac4C maintained tRNA^Ser^ stability [88]. Although the specific function of 18S rRNA acetylation is not fully understood during the early stages, it seems that the mechanism of RNA modification plays an important role in early nucleolar processing steps, which are important for the synthesis of 18S rRNA and hence for the formation of small ribosomal subunits [16] (Figure 2).

Research on ac4C follows a classic epitranscriptomics trajectory—from initial discovery to gradual elucidation of its functions and enzymatic machinery. However, the precise molecular mechanisms through which ac4C mediates these effects remain incompletely resolved.

### 3.2 Ac4C and Related Enzymes

In classical biological research, RNA modification was regulated by writers and erasers to allow dynamic changes and eventually exert function through the reader proteins [9, 89]. Currently, studies suggest that NAT10 and homologs act as the “writers” mediating ac4C modification. NAT10 belongs to the GCN5‐related N‐acetyltransferase (GNAT) family and contains 1025 amino acids with a molecular weight of 116 kDa [18]. It was a lysine acetyltransferase mediating ac4C modification of mRNA, which contained three conserved structural domains: the N‐terminal acetylase structural domain, the ATP guanosine triphosphate binding motif, and the ATPase structural domain [13, 90]. NAT10 and its homologs are highly conserved across a spectrum of organisms, including humans, mice, bacteria, and even parasites [91]. Studies suggest that in murine embryos, NAT10 is crucial for both the morula‐to‐blastocyst transition and zygotic genome activation, with NAT10 knockdown resulting in the stalling of most embryos at the morula stage [92]. NAT10 was expressed primarily in the nucleolus of cells, which could acetylate target proteins and regulate telomerase function, DNA damage response, cytokinesis, nuclear architecture, and cancer development [93, 94, 95, 96, 97]. In humans and mice, N‐acetyltransferase 10 (NAT10 and Nat10, respectively) represented the orthologs of bacterial TmcA, yeast Rra1p, and *Thermococcus kodakarensis* Nat10 (TkNat10). More recent work reveals that NAT10 also acetylates Rloops to promote their timely resolution, contributing to transcriptional regulation and genomic stability [98].

NAT10 catalyzes ac4C deposition using acetylCoA and ATP as cosubstrates, modifying specific RNA residues, including position 1842 of human 18S rRNA. Depletion of NAT10 leads to accumulation of the 30S precursor to 18S rRNA, resulting in defective ribosome assembly and impaired cellular proliferation [15]. NAT10 requires additional cofactors to introduce ac4C into diverse RNA species. For site‐specific modification of 18S rRNA, a small nucleolar RNA antisense element is necessary for target recognition. Moreover, the adapter protein THUMPD1 facilitates NAT10‐mediated acetylation within the Darm structures of tRNA^Ser^ and tRNA^Leu^ [16].

Kre33 acetyltransferase was another ac4C catalytic enzyme, interacting with the conserved adaptor *TAN1*, catalyzing the ac4C modification at the yeast tRNA^Leu^ and tRNA^Ser^ C12 sites, thereby promoting correct translation. Additionally, TmcA in bacteria and Rra1p in yeast also contributed to modifying ac4C. TmcA is a nonessential gene whose deletion did not cause overt growth defects. Compared with TmcA and NAT10, TmcAL was deficient in the helicase domain or GNAT domain, also without acetyl‐CoA as a cofactor [99]. The mechanism was similar to aminoacyl‐tRNA synthetases. Recent research has uncovered TkNat10, an RNA acetyltransferase required for archaeal thermotolerance, which is critical for the activity of the thermophilic archaeon *T. kodakarensis* under high temperatures. Unlike the eukaryotic counterparts, TkNat10 exhibited significant independent activity by modifying several RNA substrates in an ATP‐, acetyl‐CoA‐, and temperature‐dependent manner [100]. However, currently the utility of TkNat10 is constrained to instances where a consensus motif is present for precise RNA acetylation. Efforts to broaden the substrate specificity of this enzyme might be aided by directed evolution approaches in which mutations can be harnessed to bring about functional improvements in RNA or protein function.

The molecular mechanism underpinning NAT10 function is now relatively well characterized. The acetyltransferase activity of NAT10 is contingent upon substrate recognition and binding of acetyl‐CoA within an evolutionarily conserved binding pocket. This pocket facilitates stable molecular interactions—including hydrophobic contacts, hydrogen bonding, and π–π stacking—with key catalytic and structural residues. Enzymatic efficiency relies on the conservation of these residues, overall structural integrity of the binding site, and the precise geometry of ligand–residue interactions. The established NAT10 inhibitor, *Remodelin*, exerts its effect by occupying this substrate‐binding pocket, thereby competitively inhibiting acetyl‐CoA binding and diminishing acetyltransferase activity [101]. These detailed structural and mechanistic insights provide a critical foundation for the rational design of highly specific NAT10 inhibitors, including small molecules that target these conserved structural motifs for therapeutic intervention.

**FIGURE 3:**
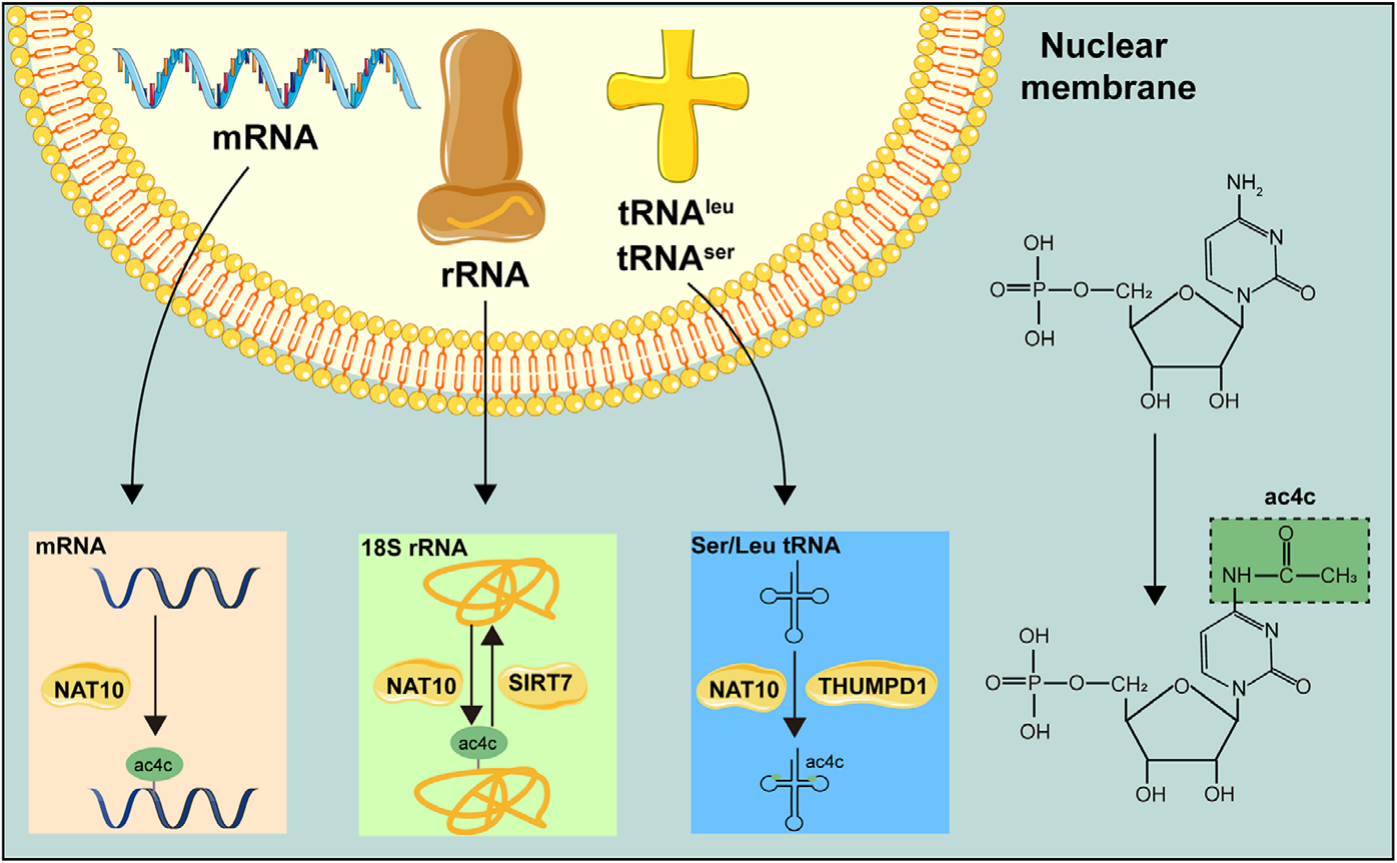
The structure of ac4C modification. NAT10‐mediated ac4C modifications are present in various RNAs and function by altering bases at different sites on the RNA or requiring other auxiliary proteins. On 18S rRNA, SIRT7 also functions as an eraser, collaborating with NAT10 to mediate the writing and erasing of ac4C modifications.

NAT10 and its evolutionary homologs are identified as the primary enzymes catalyzing ac4C formation. Early studies in this area did not find specialized eraser or reader proteins for this mark. However, this led to the discovery that sirtuin family member SIRT7 is an ac4C deacetylase on rRNA, making it a potential regulator of cellular aging [29]. So far, there have been no dedicated eraser enzymes or canonical reader proteins for ac4C on mRNA identified. As a result, the regulatory mechanisms and functional roles of ac4C in mRNA biology remain largely unclear, which is a gap in knowledge (Figure 3).

Elucidating the precise biological functions of RNA modifications forms a critical foundation for the development of targeted clinical interventions. It is imperative to systematically clarify the distinct roles these modifications play across diverse physiological and pathological states, which will be essential for advancing their potential as therapeutic targets.

## 4 The Role of RNA Modifications in Pathological Processes: A Case Study With Ac4C as a Deep Analysis

RNA modifications exert critical functions across diverse organ systems. Exemplified by m^6^A, these modifications modulate multiple facets of gene expression, including transcriptional regulation, RNA stability, translational efficiency, and interactions with RNA‐binding proteins.

Many research have previously discussed the vital function of m^6^A. For example, m^6^A plays an important role in lipid synthesis and metabolism within hepatocytes. M^6^A affects the changes in the expression of METTL3 in hepatocytes, thereby influencing the physiological and pathological processes of nonalcoholic fatty liver disease and nonalcoholic steatohepatitis [102, 103]. Furthermore, m^6^A affects oncogene and tumor suppressor gene expression and regulates liver cancer cell proliferation, migration, and invasion. Blocking m^6^A‐related enzymes may provide new intervention strategies for liver cancer [104]. M^6^A modification is thought to be associated with heart function and related diseases in the cardiovascular system. The survival and function of cardiomyocytes are affected by m^6^A modification. M^6^A methylation is involved in the production of inflammation‐related cytokines during the inflammatory process of cardiogenic shock and the functional state of the myocardium [105]. M^6^A is also gaining recognition for its regulatory role in the nervous system. Neuronal development, synaptic plasticity, and the occurrence of neurodegenerative diseases are influenced by m^6^A modification. For example, neurological diseases like Alzheimer's disease (AD) show irregular m^6^A levels, which can be linked to the disease pathology. M^6^A can modulate the activity and health of neural cells by regulating gene translation involved in neural development and inflammatory activity [9]. Generally, RNA modifications widely influence the physiological functions and pathological processes of different organ systems. They are essential to nearly all vital activities of life in organisms, from regulating gene expression and affecting cell fate to the occurrence and development of diseases. We will use ac4C modification as a case study for analysis and discuss the important role RNA modifications, represented here by ac4C, play in life activities.

Previous research suggested that ac4C is mainly present in tRNA and rRNA, but Arango showed that ac4C is also present in mRNA [13, 106]. In addition, the presence of the ac4C modification appeared to bear significant weight in the modulation of gene expression through mRNA splicing and translational machinery, playing a key role in physiological activities. The alterations occur in various disorders, including malignancies, immune disorders, skeletal balance disorders, neuropsychological disorders, heart disorders, metabolic disorders, and more.

### 4.1 Cancers

The RNA modification enzymes acted as holoenzymes, made up of the catalytic subunit joined with a regulatory RNA‐binding subunit [107]. NAT10 has an RNA acetyltransferase domain, and THUMPD1 has an RNA‐binding motif; both are necessary for ac4C formation in mRNA in human cells [15]. In particular, THUMPD1 functions as a specific ac4C adaptor to recruit NAT10 to catalyze RNA acetylation and form ac4C [16]. Studies reviewed the expression levels of THUMPD1 and its prognostic impact in different cancer types using The Cancer Genome Atlas (TCGA) database. High levels of THUMPD1 were found in several cancers, but each cancer has its own unique prognostic value. It is suggested that THUMPD1 may be a prognostic marker for cancer prognosis and response to immune‐based therapies in a variety of cancers [108].

Research shows that NAT10 expression is significantly higher in numerous cancer cell lines in studies assessing the role of NAT10 in cancer. Interestingly, increased expression improves the relationship with the infiltration of multiple immune cell types throughout the system [109]. Notably, NAT10 and its orchestrated ac4C modification were chiefly linked to the malignant nature of cancer, with the potential to modulate metabolic pathways within cancerous cells and tissues. Certain investigations have posited that NAT10 interacts with a mechanosensitive protein complex at the nuclear pore, susceptible to transfers, while the absence of NAT10 was implicated in the misplacement of p300 toward heterochromatin domains. Depleting NAT10 disrupts enhancer function, which leads to abnormal transcription of metastasis‐promoting genes, such as decreased recruitment of myeloid cells to chemokines, and hence impaired tumor metastasis [110]. In the realm of energy metabolism, Mahmood Hassan Dalhat observed that NAT10 has the capacity to govern fatty acid metabolism and stimulate ferroptosis in cancer cells through ac4C modifications [110, 111]. The reduction in ac4C levels accelerated the degradation rate of GCLC and SLC7A11 mRNA. This resulted in a decrease in intracellular cystine levels, a decrease in glutathione (GSH) production, a decrease in detoxification of reactive oxygen species (ROS), and an increase in cellular oxidized phospholipids. These cascading effects ultimately fostered the initiation of ferroptosis in cancer cells [111].

#### 4.1.1 Colorectal Cancer

Colorectal cancer (CRC) stands as the fifth most frequently occurring malignancy on a global scale [112, 113]. Metastasis stands as the primary cause of death in CRC. Unfortunately, patients with metastatic CRC currently lack truly effective therapeutic options [114]. Current evidence indicates that NAT10 exhibits elevated expression levels in colon cancer tissues as well as in multiple colon cancer cell lines [115]. Experimental work shows that silencing NAT10 in two colon cancer cell lines, HT‐29 and LoVo, effectively suppresses their ability to proliferate, migrate, invade, form tumors, and metastasize [116]. In LoVo cells specifically, NAT10 appears to suppress ferroptosis in CRC cells by preserving the mRNA stability of ferroptosis suppressor protein 1 (FSP1), a recognized GSH‐independent inhibitor of ferroptosis [117]. Additional studies demonstrate that NAT10 mediates ac4C modification on NANOGP8 mRNA, which increases mRNA stability and promotes cancer stem cell‐like characteristics. These effects consequently diminish cellular responsiveness to conventional chemotherapy [118].

#### 4.1.2 Osteosarcoma

Osteosarcoma represents the most prevalent primary malignant bone tumor in children and adolescents and is associated with substantial morbidity and mortality [119]. While the adoption of neoadjuvant chemotherapy combined with surgical intervention has substantially improved the 5‐year overall survival rate, approximately one‐third of patients still experience recurrence and metastasis during treatment, culminating in adverse outcomes [120, 121, 122, 123]. Consequently, elucidating the molecular mechanisms driving osteosarcoma progression is critical for identifying novel therapeutic targets and developing effective pharmacological agents. Although prior studies have established the importance of RNA modifications in osteosarcoma pathogenesis, the role of ac4C modification remains largely unexplored [124]. In recent years, NAT10 has garnered increasing attention in the medical literature. Elevated NAT10 expression correlates with poor prognosis in osteosarcoma patients and has been shown to promote tumor proliferation and metastasis. Mechanistically, ac4C modification facilitates the enhancement of mRNA stability and translational efficiency of downstream target genes [125]. Specifically, NAT10 stabilizes activating transcription factor 4 (ATF4) mRNA through ac4C modification, leading to upregulation of asparagine synthetase (ASNS) and subsequent asparagine biosynthesis, which drives the development of osteosarcoma. Additionally, NAT10‐induced ac4C on YTHDC1 mRNA stabilizes it and upregulates its expression. Subsequent investigation reveals that YTHDC1 can recognize m^6^A sites on phosphofructokinase (PFKA) and lactate dehydrogenase A (LDHA). These two enzymes are essential for the glycolytic pathway. This allows m^6^A‐dependent stabilization of their transcripts and heightened glycolytic flux that can support tumor growth [125]. Gao et al. demonstrated that inhibition of ac4C acetylation in osteosarcoma impedes proliferative and metastatic capacities while inducing apoptosis and cell‐cycle arrest [126]. *Remodelin* was found to be a specific inhibitor of NAT10. They further utilized network pharmacological analysis to identify five genes, CASP3, ESR2, FGFR2, IGF1, and MAPK1, as key therapeutic targets of *Remodelin* against osteosarcoma [127].

#### 4.1.3 Esophageal Squamous Cell Carcinoma

Esophageal squamous cell carcinoma (ESCC) represents a predominant histological subtype of esophageal cancer, a disease widely recognized as one of the most aggressive and highly metastatic malignancies characterized by a poor overall prognosis [128]. Long Liao and colleagues discovered that among the newly identified lysine acylation modifications, the level of lysine 2‐hydroxyisobutyrylation (Khib) showed significant differences in highly metastatic ESCC cell sublines and in metastatic tumor tissues. Furthermore, NAT10 was found to promote the Khib modification specifically at the 823‐lysine site by enhancing ac4C‐dependent NOTCH3 mRNA stability, thereby facilitating tumor metastasis [129]. RNA‐binding proteins, which attach to specific RNA molecules to form ribonucleoprotein complexes, have their interactions with various biological macromolecules modulated through long noncoding RNAs (lncRNAs) that function as scaffolds or decoys [1, 130]. The NAT10‐mediated ac4C modification may contribute to the overexpression of lncRNA CTC‐490G23.2 observed in primary ESCC, where this lncRNA functions as a scaffold that binds CD44 pre‐mRNA to polypyrimidine tract binding protein 1 (PTBP1), ultimately resulting in malignant splicing that switches the standard CD44s isoform to the variant isoform CD44v, thereby promoting cancer metastasis [131]. NAT10 could additionally modulate macrophage lipid metabolism and polarization within the ESCC microenvironment. METTL3 was shown to enhance NAT10 expression by facilitating m^6^A modifications in the 3′ UTR of NAT10. NAT10 promoted expression of fatty acid synthase through ac4C modifications, which in turn fostered M2 polarization of macrophages by mediating lipid metabolism [132]. Besides, other studies have suggested that clinical knockdown of NAT10 using gefitinib could potentially inhibit esophageal cancer progression [133].

#### 4.1.4 Gastric Cancer

Gastric cancer (GC) represents a widespread malignancy characterized by both high incidence and mortality rates [128]. The investigation further revealed that NAT10 expression and ac4C levels are notably heightened in GC. NAT10 facilitates GC progression by orchestrating ac4C alterations in LDHA1 mRNA and programming Hexokinase 2 mRNA to enhance its stability, thereby activating the glycolytic pathway [134]. NAT10 additionally promotes glycolytic addiction by mediating ac4C modification of SEPT9 mRNA, which hyperactivates the HIF‐1α pathway and rewires glucose metabolism [135]. Advanced GC frequently leads to distant metastasis, which contributes to a suboptimal 5‐year survival rate [136]. Recently, neutrophil extracellular traps (NETs) have been implicated as promoters of cancer metastasis [137]. Scientists suggested that exposure to NETs can promote NAT10‐mediated ac4C modification of SMYD2 mRNA, which stabilizes the mRNA and enhances the metastatic potential of GCs [138]. Downregulation of NAT10 can reduce global ac4C levels in GC, thereby inhibiting AKT phosphorylation and epithelial–mesenchymal transition (EMT). This significantly suppresses the proliferation, migration, invasion, and cell‐cycle progression of GCs [139].

#### 4.1.5 Lung Cancer

Lung cancer ranks as the second most frequently diagnosed cancer worldwide. Among its various histological subtypes, lung adenocarcinoma (LUAD) has now surpassed squamous cell carcinoma in prevalence [128]. Studies have shown that LUAD exhibits markedly higher levels of ac4C modification and NAT10 expression compared to adjacent nontumor tissues. In terms of mechanism, the NAT10/THUMPD1 complex is responsible for catalyzing ac4C modification on primary microRNA (pri‐miRNA) transcripts. This acetylation, in turn, facilitates the processing of pri‐miRNA into precursor miRNA (pre‐miRNA) by strengthening the interaction between pri‐miRNA and the DGCR8 microprocessor subunit, thereby augmenting the biogenesis of mature miRNA [140].

Non–small‐cell lung cancer (NSCLC) is the most common type of lung cancer. However, NAT10 has a dual role in the progression of NSCLC. On one hand, NAT10 maintains glycolytic flux and inhibits apoptosis in NSCLC cells by ac4C modification of ENO1 mRNA [141]. Pharmacological inhibition of NAT10 with *Remodelin* inhibits NSCLC proliferation, invasion, and migration through modulation of the EMT pathway [142]. On the other hand, NAT10 upregulates SGK2 mRNA stability via ac4C modification in its 3′ UTR, increasing the level of autophagy in NSCLC cells [143].

#### 4.1.6 Breast Cancer

Breast cancer (BC) is a diverse cancer with increasing global incidence. LncRNAs have been implicated in the development and progression of BC. Bioinformatics analyses have established that the lncRNA CD2BP2‐DT is overexpressed in BC; it correlates with worse clinicopathological features and poor survival. Mechanistically, NAT10 promotes deposition of ac4C modification on CD2BP2‐DT, which increases RNA stability and cellular abundance. Subsequently, the liquid–liquid phase separation of YBX1 stabilized by CD2BP2‐DT is essential for CDK1 mRNA. This regulatory axis leads to increased BC cell proliferation and tumor growth [144].

Triple‐negative breast cancer (TNBC) is an aggressive molecular subtype defined by the lack of estrogen receptor (ER), progesterone receptor (PR), and human epidermal growth factor receptor 2 (HER2) expression, for which therapeutic options remain limited and often yield suboptimal outcomes [145]. NAT10 was highly expressed in TNBC and BC [146]. Bioinformatic investigations have identified three prognostic risk lncRNA pairs whose expression is influenced by NAT10 activity, specifically the reciprocal pairs “LINC01614‐COL3A1,” “OIP5‐AS1‐USP8,” and “RP5‐908M14.9‐TRIR” [147]. It is postulated that inhibition of NAT10 would attenuate ac4C deposition on transcripts encoding ATP‐binding cassette (ABC) transporters, including multidrug resistance protein 1 (MDR1) and breast cancer resistance protein (BCRP), thereby potentially reversing chemoresistance [146]. Furthermore, NAT10 deficiency has been shown to remodel the tumor microenvironment in TNBC by constraining glycolytic metabolism while simultaneously promoting T‐cell infiltration and activation, thereby sustaining an antitumor immune state [148]. Reflecting the role of NAT10 in promoting oncogenic traits, a recently characterized circular RNA derived from the peptidylprolyl isomerase D (PPID) locus has been identified as a suppressor of trastuzumab resistance. This circRNA directly interacts with and physically sequesters NAT10 in the nucleus, disrupting its association with HER2 mRNA. Consequently, it diminishes ac4C modification specifically within exon 25 of HER2, thereby attenuating HER2 mRNA stability and protein expression. This mechanism effectively resensitizes BC cells to trastuzumab treatment [149].

#### 4.1.7 Cervical Cancer

Cervical cancer (CCa) stands as the fourth most prevalent malignant disease worldwide [150, 151]. The expression level of NAT10 is markedly elevated in CCa tissues. The transcription factor HOXC8 activates NAT10, which in turn enhances the ac4C modification on FOXP1 mRNA. This modification increases the translational efficiency of FOXP1 and subsequently upregulates downstream metabolic effectors, including GLUT4 and ketohexokinase (KHK), thereby promoting glycolytic flux and lactate production in CCa cells [152]. Additionally, NAT10 is responsible for the ac4C modification of HNRNPUL1 mRNA and solute carrier family 7 member 5 (SLC7A5) mRNA. This modification enhances mRNA stability and increases protein expression, which contributes to promoting CCa cell proliferation, invasion, and migratory capacity [153, 154]. Therefore, targeted inhibition of NAT10 can serve as an effective way to slow down CCa progression. Research teams have identified CircMAST1 as a tumor‐suppressing RNA that competes with NAT10 for binding. This competitive interaction blocks NAT10 from adding ac4C marks to YAP mRNA, leading to breakdown of that transcript and consequently restraining CCa advancement [155].

#### 4.1.8 Bladder Urothelial Carcinoma

Bladder cancer (BLCA) stands out as one of the most frequently occurring malignant tumors that affect the urinary system [151]. NAT10 and ac4C modification may be the molecular targets for therapy. When NAT10 expression is reduced, the precise ac4C modification on key transcripts becomes compromised, which in turn obstructs the translation of important proteins such as BCL9L, SOX4, and AKT1, while the concurrent destabilization of BCL9L and SOX4 mRNA transcripts serves to suppress the cancer‐forming abilities of bladder tumor cells [156]. Additionally, the chemotherapeutic cisplatin, a BLCA drug, activates the NF‐κB pathway. Transactivation of the NAT10 promoter by the NF‐κB p65 subunit thus activates NAT10 expression. NAT10 stabilizes AHNAK mRNA and protects it from degradation by exonucleases, which facilitates DNA damage repair and promotes cisplatin chemoresistance [157].

#### 4.1.9 Hepatocellular Carcinoma

Hepatocellular carcinoma (HCC) represents the most frequently occurring histological subtype of liver cancer, arising from a complex interplay of multiple etiological factors [158]. Global upregulation of RNA ac4C and the writer enzyme NAT10 that generates this mark is associated with poor prognosis in HCC patients [159]. Multiple lines of investigation have demonstrated that high‐mobility group protein B2 (HMGB2) functions as an oncogenic driver and actively contributes to the advancement and progression of HCC [160]. NAT10 enhances the translational efficiency of HMGB2 mRNA through the deposition of ac4C modifications within its coding sequence, which in turn fortifies the interaction between the mRNA and eukaryotic elongation factor 2 (eEF2) [159]. Interestingly, the role of ac4C modification exhibits transcript‐specific duality in HCC. It facilitates infiltration of M1 macrophages and reduces myeloid‐derived suppressor cell (MDSC) infiltration in the tumor immune microenvironment, while upregulating the oncogene HMGB2. Blocking ac4C hinders PD‐L1 mRNA decay in myeloid cells and impairs cytotoxic T lymphocyte‐mediated tumor elimination [161]. Through comprehensive high‐throughput screening of chemical compounds, researchers successfully identified the HDAC inhibitor Panobinostat as a potent suppressor of NAT10‐mediated ac4C modification activity [159]. Furthermore, combined administration of the NAT10 inhibitor *Remodelin* alongside the PARP inhibitor Olaparib has demonstrated synergistic antitumor effectiveness specifically against cancers that have developed resistance to PARP inhibition [162].

#### 4.1.10 Pancreatic Ductal Adenocarcinoma

Pancreatic ductal adenocarcinoma (PDAC) is the 12th most common cancer in the world and is the seventh leading cause of cancer death. It has an extremely poor 5‐year survival rate of around 10% [151]. Clinical management of PDAC is a significant therapeutic challenge. Gemcitabine resistance in PDAC has recently been associated with heightened NAT10 expression [163]. Given the potential for cure, surgical resection typically forms part of a multimodal treatment approach. NAT10 activation of the TGF‐β signaling pathway enhances tumor vascularization and promotes distant metastasis. Consequently, the migration and clonogenic ability of PDAC cells is severely compromised by genetic or pharmacological inhibition of NAT10 [164]. Mechanistically, NAT10 enhances the stability of AXL mRNA in an ac4C‐dependent manner, which in turn causes the expression of the AXL receptor tyrosine kinase that enacts potent oncogenic functions to promote PDAC proliferation and metastasis [165].

#### 4.1.11 Ovarian Cancer

Ovarian cancer (OC) has the highest mortality rate among gynecological cancers [151]. NAT10 actively promotes ovarian cancer cell migration, invasion, and the maintenance of stemness properties through an ac4C‐mediated upregulation of CAPRIN1 expression levels [166]. It is important to note that a functionally relevant crosstalk exists between ac4C and m^6^A modifications during OC progression. Specifically, METTL14‐mediated m^6^A methylation modifies NAT10 mRNA translation through the reader protein IGF2BP1, thereby enhancing the expression of NAT10. Then, NAT10 can enhance the stability and translation efficiency of ACOT7 mRNA by catalyzing ac4C modification, thereby altering the fatty acid metabolism of cancer cells and promoting their survival. Fortunately, the nucleoside analog fludarabine effectively inhibits this pathway [167].

#### 4.1.12 Nasopharyngeal Carcinoma

Nasopharyngeal carcinoma (NPC) is one of the most common malignancies of the head and neck region [168]. Eukaryotic translation elongation factor 1A2 (eEF1A2) is a GTPase which possesses oncogenic properties [169, 170, 171]. In NPC, the lncRNA SIMALR is a highly abundant cytoplasmic lncRNA. The ac4C modification of SIMALR enhances its interaction with eEF1A2, thereby promoting eEF1A2 phosphorylation and increasing the translation efficiency of downstream target genes ITGA6 and ITGB4, which facilitates NPC proliferation and migration [172]. The combination of NAT10 inhibitor *Remodelin* and sorafenib can suppress ac4C‐modification‐mediated upregulation of solute carrier family 7 member 11 (SLC7A11) expression, thereby facilitating the normal progression of ferroptosis in NPC cells [173].

One major mechanism of immune evasion in NPC is immunosuppression, especially T‐cell dysfunction. NAT10‐catalyzed ac4C modification strengthens CEBPG, DDX5, and HLTF mRNAs, augmenting their translation. The NAT10/ac4C/DDX5 axis promotes expression of high‐mobility group box 1 (HMGB1) and inhibits CD4^+^ and CD8^+^ T‐cell activity, thereby establishing an immunosuppressive tumor microenvironment [174].

#### 4.1.13 Oral Squamous Cell Carcinoma

Oral squamous cell carcinoma (OSCC) is the most common malignancy in the head and neck. It comprises over 90% of all oral cancer cases [151]. Research has shown that NAT10 is much more prevalent in tissues with OSCC compared to normal oral tissues. The overall levels of MMP1 mRNA and its ac4C acetylation were substantially decreased when NAT10 was knocked down, which caused a decrease in mRNA stability. Further xenograft assays confirmed the reduction of MMP1 expression in vivo and additionally demonstrated that NAT10 knockdown could inhibit the tumorigenicity and metastatic ability of OSCC cells [175].

#### 4.1.14 Prostate Cancer

Prostate cancer arises as a malignancy from the prostate gland within the male reproductive system, which has the capacity to progress aggressively and metastasize to distant organs [151, 176]. In the context of prostate cancer, NAT10 expression is upregulated, which in turn enhances tumor cell proliferation and invasive capacity [177]. NAT10 stabilizes HMGA1 and KRT8 mRNAs by ac4C modification, which promotes cell‐cycle progression and EMT, thereby driving tumor growth and migration [178]. NAT10 also contributes to the creation of an immunosuppressive tumor microenvironment by impairing both the recruitment and cytotoxic activity of CD8^+^ T cells through the CCL25/CCR9 axis [177].

#### 4.1.15 Skin Cancer

Ultraviolet B (UVB) radiation constitutes a segment of the solar spectrum capable of penetrating the epidermal layer, thereby serving as a primary environmental carcinogen responsible for elevated risk of cutaneous malignancies [179]. Among the most genotoxic UVB‐induced DNA lesions are cyclobutane pyrimidine dimers (CPDs), which represent critical initiating events in skin carcinogenesis. In human HaCaT keratinocytes, knockdown of NAT10 substantially decreased global ac4C levels and accelerated the repair rate of UVB‐induced CPDs [180]. The global genome nucleotide excision repair (GG‐NER) pathway is the principal mechanism for recognizing and repairing such helix‐distorting DNA lesions [181]. Investigation into this phenotype revealed that NAT10 depletion specifically upregulated the protein expression of two key GG‐NER factors, XPA and DDB2, without altering the levels of XPB, XPC, XPD, XPF, and XPG [180].

#### 4.1.16 Melanoma

Melanoma is a type of skin cancer originating from melanocytes, which commonly uses dacarbazine (DTIC) as a treatment [182, 183]. The Cys2His2 (C2H2) zinc‐finger family constitutes the largest group of transcription factors in humans; among its members, DDX41 and ZNF746 have been implicated in promoting oncogenic progression [109]. NAT10 enhances the expression levels of DDX41 and ZNF746 through ac4C‐mediated mRNA modification, thereby contributing to increased DTIC resistance in melanoma models both in vitro and in vivo. Notably, pharmacological inhibition of NAT10 with *Remodelin* meaningfully restores cellular sensitivity to DTIC, providing a mechanistic rationale for developing novel combinatorial strategies to overcome chemoresistance in melanoma patients [184].

#### 4.1.17 Acute Myeloid Leukemia

Acute myeloid leukemia (AML) represents an aggressive hematologic malignancy that originates from leukemia‐initiating cells (LICs) [185]. NAT10 drives both the uptake of serine and its de novo biosynthesis in AML cells. This effect is achieved by boosting the translational efficiency of the serine transporter SLC1A4 and of key transcriptional regulators such as HOXA9 and MENIN. Pharmacologically blocking NAT10 through fludarabine or *Remodelin* results in the effective elimination of AML cells [186].

**FIGURE 4:**
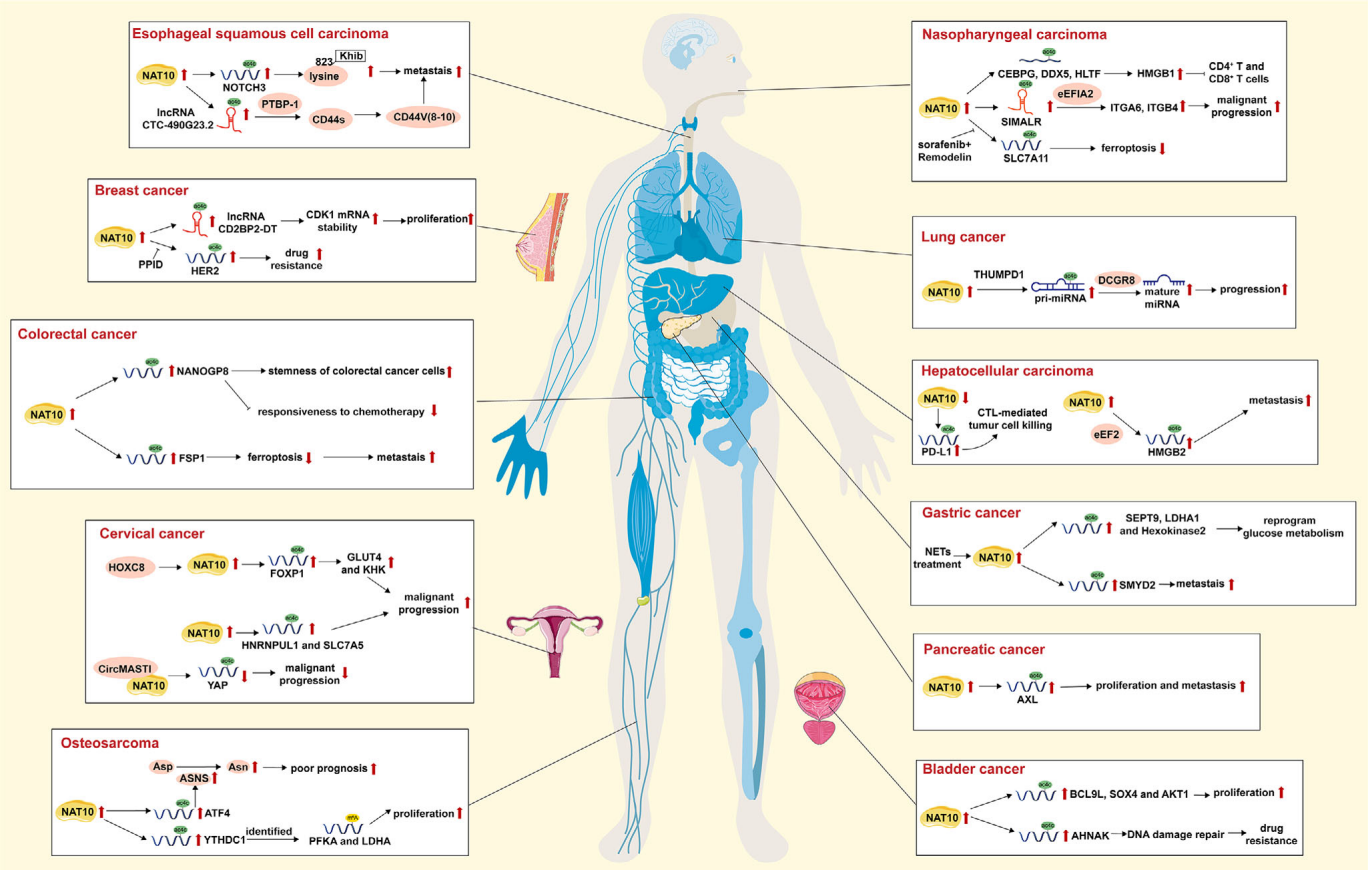
The mechanism of ac4C modification regulating tumor malignant progression. Ac4C RNA modification has been implicated in promoting tumorigenesis and disease progression across a wide spectrum of malignancies. The enzyme NAT10, which catalyzes this modification, serves as a central regulator in these pathophysiological processes, driving key oncogenic mechanisms.

NAT10 and its mediated ac4C modifications are putative targets for cancer therapy that should lead to more efficient strategies for conquering cancer in human health (Figure 4).

### 4.2 Immunological and Inflammatory Disorders

The immune system functions as an intricate network made up of various cells, tissues, and organs that work together to shield the body from dangerous invaders [187]. This elaborate defense mechanism plays a fundamentally important role in maintaining the overall health of any individual.

Systemic lupus erythematosus (SLE) is a fairly complex autoimmune disorder that presents with diverse clinical manifestations, where pathogenic autoantibodies and chronic inflammation serve as the primary mechanisms [188]. Recent research also suggests that post‐transcriptional RNA modifications—particularly ac4C deposition—contribute to autoimmune pathogenesis, mainly by altering how CD4+ T cells function [189]. Transcriptome‐wide mapping in SLE reveals widespread ac4C modification changes, with affected mRNAs showing significant enrichment in key biological processes like metabolic adaptation, oxidative stress responses, apoptotic regulation, and NF‐κB signal transduction [190].

Viral pathogens are capable of triggering dynamic shifts in the host's RNA modification landscape. When cells are infected with influenza A virus in vitro, NAT10 expression levels tend to decrease [191]. However, during enterovirus 71 (EV71) infection, the NAT10 enzyme derived from the host mediates the incorporation of ac4C modifications into the viral RNA genome at the 5′ untranslated region [192]. An EV71 mutant that cannot perform ac4C modification showed significantly reduced virulence, which highlights ac4C as a promising target for antiviral strategies [193].

Naive T‐cell activation demands substantial proteomic remodeling, which is supported by increased protein synthesis [194]. Upon T‐cell activation, NAT10 is upregulated, thereby enhancing Myc translation through ac4C modification to drive Alzheimer’s disease (AD) is vigorous proliferative responses. Conditional deletion of NAT10 in murine T cells severely impairs cell‐cycle progression and expansion due to insufficient MYC expression, leading to worse outcomes in acute lymphocytic choriomeningitis virus infection [195].

Sepsis‐associated pulmonary injury (SPI) constitutes a fairly common and particularly serious complication that substantially contributes to sepsis‐related fatalities [196]. Emerging evidence increasingly indicates that ferroptosis actively participates in SPI pathogenesis. More specifically, NAT10‐mediated ac4C modification serves to stabilize TFRC mRNA, thereby increasing transferrin receptor expression. This upregulation subsequently facilitates cellular iron accumulation and lipid peroxidation, which in turn promotes ferroptotic death in pulmonary endothelial cells and aggravates lung damage [197]. Pyroptosis also makes a significant contribution to tissue injury during sepsis. When NAT10 was specifically overexpressed in neutrophils through transgenic methods, septic mice showed improved survival rates and less severe pulmonary damage, mainly because pyroptotic cell death was suppressed. In contrast, inhibiting NAT10 lowered ULK1 expression, which in turn amplified the STING—activity of the IRF3 pathway, assembly of the NLRP3 inflammasome, and ultimately pyroptosis in neutrophils [198].

**FIGURE 5:**
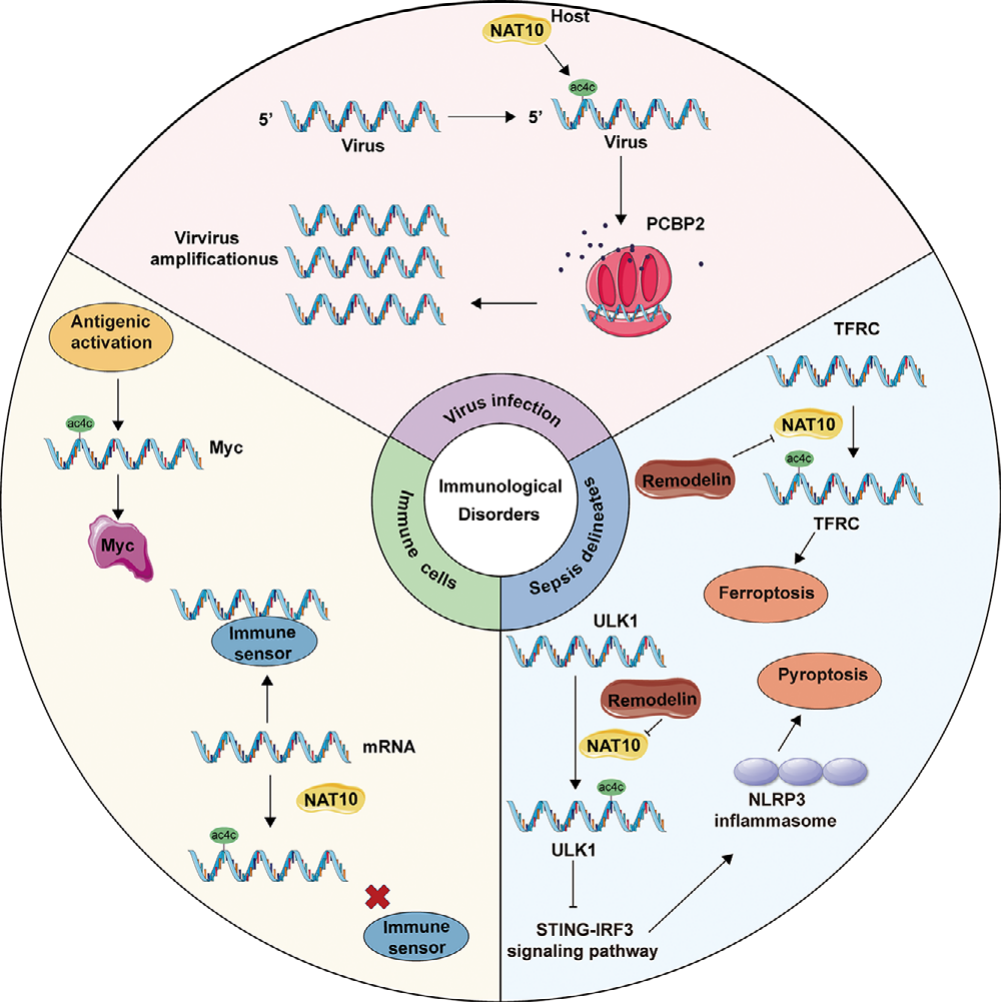
The involvement of ac4C in the immune system. Following viral infection, NAT10 catalyzes ac4C deposition on viral mRNAs, a process that promotes their stability and translational efficiency, ultimately augmenting viral replication. In the context of systemic lupus erythematosus, NAT10 upregulates ULK1, which in turn attenuates the activation of the STING‐IRF3 signaling axis, leading to suppressed NLRP3 inflammasome activity within neutrophils. Furthermore, the incorporation of ac4C in place of cytidine in synthetic mRNA constructs diminishes the induction of inflammatory genes in immune cells by reducing ligand recognition by innate immune sensors. During T‐cell activation, elevated levels of ac4C modification are observed on Myc mRNA, suggesting a role in modulating the adaptive immune response.

Nevertheless, NAT10's role in sepsis progression appears context‐dependent. Pharmacological inhibition of NAT10 with compounds such as *Remodelin* can mitigate injury associated with NAT10 activity. In rodent models of sepsis, *Remodelin* administration alleviated characteristic pathological features, including pronounced infiltration of erythrocytes and inflammatory cells into alveolar spaces, marked thickening of alveolar septa, and partial alveolar collapse [197]. Furthermore, incorporation of ac4C in place of cytidine in synthetic mRNA constructs suppressed inflammatory gene expression in immune cells. This chemical substitution also altered the mRNA–protein interactome by reducing binding to cytidine‐specific RNA‐binding proteins and an innate immune sensor, suggesting that ac4C incorporation could optimize nucleic acid therapeutics for improved efficacy [199] (Figure 5).

### 4.3 Neuropsychiatric Disorders

Vision entails a sophisticated process orchestrated through the intricate workings of the nervous system [200]. Ac4C modifications have been identified to affect the recovery from corneal cold damage. Mesenchymal stem cells derived from murine amniotic fluid were observed to foster mRNA ac4C alterations and heightened NAT10 expression in ocular tissues, concomitant with a markedly elevated ETV4/JUN/CCND2 signaling cascade [201]. However, ac4C does not have a clear function in all diseases. The expression of ac4C was detected in the serum of uveitis patients in a study by Lei Feng et al., but there was no significant difference between healthy controls and patients with uveitis [202].

Moreover, the precise spatial patterning of ac4C is critical for normative brain development. acRIP‐seq analyses revealed that ac4C peaks are predominantly enriched in the thalamus, primarily within 3′ UTR and CDSs. Gene Ontology (GO) and Kyoto Encyclopedia of Genes and Genomes (KEGG) pathway analyses of differentially acetylated transcripts revealed significant enrichment within key neuroinflammatory and developmental signaling pathways, including NF‐κB, TNF, and Toll‐like receptor cascades. Intriguingly, NAT10 was found to mediate central neuropathic pain following thalamic hemorrhage (TH) by upregulating Fn14 expression via the NF‐κB pathway; however, the precise involvement of ac4C modification in this specific regulatory axis remains to be elucidated [203].

Ischemic stroke, a neurological disease, stands as the predominant factor behind enduring and incapacitating motor and neurophysiological impairments in adults globally [204]. NAT10 is enriched in the affected cortical areas of patients with acute ischemic stroke and in the peri‐infarct cortex of mice after photothrombotic stroke. The malfunctioning of the brain can lead to a stroke. A key factor in this process is autophagy in the peri‐infarct area. NAT10 promotes the ac4C acetylation of inflammatory cytokine tissue inhibitor of metalloproteinase 1 (TIMP1) mRNA transcript and upregulates TIMP1 expression. This results in the accumulation of microtubule‐associated protein 1 light chain 3 (LC3) and the progression of astrocyte autophagy [205].

Central poststroke pain (CPSP), a neuropathic condition characterized by chronic sensory abnormalities and pain following either hemorrhagic or ischemic stroke, represents a significant clinical consequence of cerebrovascular injury [206]. Investigations into TH models have identified a marked upregulation of NAT10 expression, with immunolocalization studies indicating its predominant presence within neuronal populations [203]. In a distinct clinical context, disruptions in tRNA acetylation pathways underscore the critical importance of this modification for neurological development. Specifically, mutations in THUMPD1, a gene encoding an essential cofactor for NAT10‐mediated tRNA acetylation, are linked to a syndromic intellectual disability. This disorder presents with global developmental delay, behavioral anomalies, sensorineural hearing loss, and characteristic craniofacial features [207], thereby establishing a clear connection between defective ac4C deposition and aberrant neurodevelopment.

Epilepsy is a fairly common chronic neurological disorder that develops when the brain's threshold for producing spontaneous seizures becomes pathologically reduced [208]. The brain‐derived neurotrophic factor (BDNF), induced as an immediate–early gene, serves as a sensitive molecular marker of heightened neuronal activity during epileptogenesis [209]. NAT10 expression and global ac4C content become elevated within brain tissues. This increase in ac4C modification correlates with augmented translational output of BDNF protein, which subsequently exacerbates neuronal hyperexcitability and intensifies seizure severity [210].

The epitranscriptomic mark ac4C has been connected to fundamental neural processes [211]. Following peripheral nerve injury, vascular endothelial growth factor A (VEGFA) expression increases in the spinal cord, and inhibiting it reduces pain hypersensitivity [212]. Mechanistically, injury upregulates NAT10, which adds ac4C marks to VEGFA mRNA. This modification enhances VEGFA translation by promoting polyribosome recruitment, increasing VEGFA protein levels and facilitating central sensitization. A positive feedback loop involving HNRNPK further amplifies this process [213].

Alzheimer's disease (AD) is a progressive neurodegenerative disease clinically defined by progressive cognitive impairment. Through protein–protein interaction (PPI) network analysis, Yanzhen Ma and colleagues identified 37 hub genes potentially linking AD to dysregulated GABAergic synapses and the PI3K/AKT signaling pathway, though their precise mechanistic roles require further functional validation [214]. Additionally, bioinformatic screening has identified a recurrent CXX motif within AD‐associated lncRNAs and highlighted three specific transcripts; however, the molecular mechanisms through which these lncRNAs contribute to AD pathogenesis remain to be elucidated [215]. Notably, emerging evidence indicates that aberrant ac4C modification patterns disrupt mRNA translation and proteostatic equilibrium in the hippocampus of early‐stage AD mouse models. This dysregulation presents a promising novel avenue for the early detection and potential therapeutic intervention in AD [216].

**FIGURE 6:**
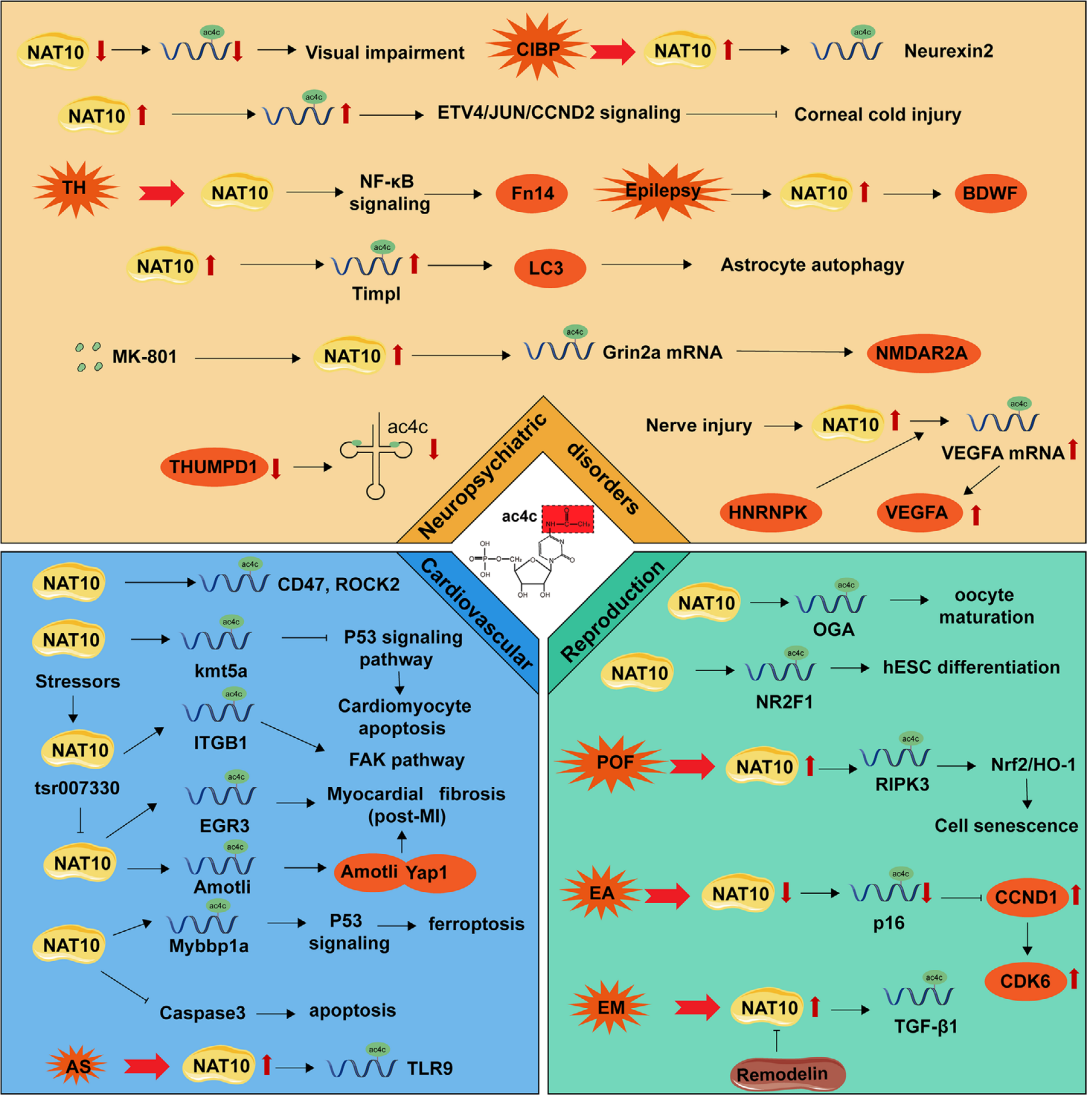
The involvement of ac4C in multiple pathological systems, including neuropsychiatric disorders, cardiovascular disease, and the reproductive endocrine system. Neuropsychiatric disorders: The depletion of NAT10 caused a reduction in the levels of ac4C on mRNA of visual proteins. The reduction in mRNA stability then led to visual defects. A study has shown that mesenchymal stem cells derived from murine amniotic fluid can enhance ETV4/JUN/CCND2 signaling to help repair injured tissue in lab mice. Moreover, it can alter mRNA ac4C as well. NAT10 increased Fn14 via NF‐κB to modulate central pain following TH. In the presence of MK‐801, NAT10 enhanced translation efficiency by increasing ac4C modifications of Grin2a mRNA, leading to an increase in NMDAR2A protein levels. THUMPD1 loss resulted in the absence of tRNA acetylation. Nerve injury did cause the expression of NAT10, which mediated the ac4C modifications on Vegfa mRNA to promote translation. HNRNPK was associated with Vegfa mRNA and recruited NAT10. NAT10 enhanced ac4C acetylation of Timp1 mRNA to upregulate TIMP1 for LC3 accumulation and astrocyte autophagy. In epilepsy, increased NAT10 enhanced BDNF expression. Cardiovascular disease: NAT10 contributes to cardiovascular pathophysiology through multiple mechanisms involving RNA acetylation. Specifically, it enhances the stability and translational efficiency of CD47 and ROCK2 transcripts via ac4C modification, thereby influencing key cellular processes. Additionally, NAT10‐mediated ac4C deposition on Kmt5a mRNA suppresses the p53 signaling pathway, reducing cardiomyocyte apoptosis. Under stress conditions, NAT10 expression is elevated, which stabilizes ITGB1 mRNA and subsequently activates FAK‐dependent signaling. Conversely, inhibition of NAT10 with tsr007330 in rat models reduces ac4C modification on EGR3 mRNA, attenuating myocardial fibrosis after myocardial infarction. In post‐MI mice, NAT10 strengthens Amotl1 mRNA stability, promoting Amotl1‐Yap1 interaction and nuclear translocation of Yap, which drives fibrotic progression. Furthermore, the NAT10/Mybbp1a/p53 axis facilitates ferroptosis in cardiomyocytes, whereas in cardiac fibroblasts, NAT10 activity inhibits caspase‐3 and attenuates apoptosis. Beyond these roles, NAT10 participates in reprogramming serine metabolism. Clinically, elevated NAT10 in atherosclerosis patients increases TLR9 ac4C modification, exacerbating disease severity. In models of premature ovarian failure, NAT10 stabilizes RIPK3 mRNA through ac4C modification, leading to activation of the Nrf2/HO‐1 pathway and accelerating ovarian senescence. Reproductive endocrine system: NAT10‐mediated ac4C modification regulated OGA expression to affect oocyte maturation and promoted ectoderm differentiation of hESC via acetylating NR2F1 mRNA. Under the condition of EA, NAT10 modulated the P16/CDK6/CCND1 axis activity in ovarian granulosa cells. In EM, ac4C modification stabilizes TGFB1 mRNA to enhance its expression, while NAT10 inhibition by *Remodelin* suppresses ectopic lesion proliferation.

Schizophrenia is a highly heritable psychiatric disorder associated with significant personal and societal burden [217]. Acute administration of MK‐801, a noncompetitive N‐methyl‐D‐aspartate (NMDA) receptor antagonist, can induce schizophrenia‐like behavioral deficits in juvenile male mice. Notably, rare variants in GRIN2A—the human ortholog of the murine Grin2a gene encoding the NMDAR2A subunit—confer increased genetic risk for schizophrenia [218]. Research indicates that in the presence of MK‐801, NAT10 potentiates the translation efficiency of Grin2a mRNA via ac4C modification. This leads to an accumulation of NMDAR2A protein in the prefrontal cortex (PFC) and correlates with the emergence of schizophrenia‐like phenotypes in mice [219] (Figure 6).

In summary, ac4C exerts a broad influence on the pathophysiology of various neurological and psychiatric conditions. However, the detailed molecular pathways through which it acts are still poorly understood. Investigations focused on the selective regulation of ac4C deposition on particular RNA targets could open avenues for new therapeutic interventions in these disorders.

### 4.4 Cardiovascular Disease

The ac4C modification is significantly involved in regulating multiple processes within the cardiovascular system, such as cardiac remodeling, cell death, fibrosis, and diabetic cardiomyopathy.

Around the world, cardiovascular disease (CVD) continues to be the main cause of death [220]. Atherosclerosis (AS) is a chronic, lipid‐driven inflammatory disease of the arterial wall, widely recognized as the principal underlying pathology of CVDs [221]. Studies have demonstrated a significant upregulation of NAT10 expression and a concomitant increase in global ac4C modification levels in clinical AS specimens. Mechanistically, genetic depletion of NAT10 promoted a phenotypic shift in macrophages from a proinflammatory M1 state toward a reparative M2 state, reduced ac4C modification on TLR9 mRNA, and consequently attenuated atherosclerotic plaque progression in vivo [222].

Cardiac remodeling represents a basic pathological change in heart failure. A cardiac‐specific Piwi‐interacting RNA (piRNA) called HAAPIR forms a complex with NAT10 to help deposit ac4C on Tfec mRNA. This modification increases Tfec expression, which then raises levels of the proapoptotic protein BIK, leading to cardiomyocyte apoptosis [223]. In addition, NAT10 plays a role in cardiac remodeling by acetylating and stabilizing CD47 and ROCK2 mRNAs through ac4C [224]. Knockdown of NAT10 leads to a decrease in ac4C levels on Kmt5a mRNA, thereby activating the p53 pathway and resulting in cardiomyocyte apoptosis [225].

The phenotypic shift of vascular smooth muscle cells (VSMCs) from a contractile to a proliferative state represents a key event in pathological vascular remodeling during CVD progression [226]. When stress or injury occurs, high levels of NAT10 bind to ITGB1 and acetylate its mRNA, making it more stable and subsequently activating FAK signaling pathways. This encourages a synthetic VSMC phenotype [227].

Myocardial infarction (MI) involves the irreversible necrosis of cardiomyocytes that occurs when coronary blood supply is abruptly interrupted [228]. In infarcted myocardial tissue, NAT10 expression shows a substantial increase. NAT10 facilitates an elevation in the ac4C modification of Amotl1, which subsequently enhances mRNA stability. This augmented interaction between Amotl1 and Yes‐associated protein 1 (YAP1) promotes the nuclear translocation of YAP1, thereby driving the fibrotic expansion of post‐MI injury [229]. Furthermore, a deleterious positive feedback loop involving NAT10, Mybbp1a, and p53 actively facilitates ferroptosis in cardiomyocytes, thereby exacerbating myocardial damage induced by ischemia–reperfusion injury [230]. Inhibiting NAT10 would lead to decreased ac4C deposition on early growth response 3 (EGR3) mRNA, which in turn reduces EGR3 protein levels and helps mitigate myocardial fibrosis after infarction. Interestingly, a counter‐regulatory tRNA‐derived small RNA known as tsr007330 becomes downregulated following MI. When tsr007330 is experimentally restored in rat hearts, it curbs NAT10 activity and improves cardiac function [231].

These findings elucidate an epitranscriptomic regulatory axis in CVD pathogenesis and may inform the development of novel therapeutic strategies targeting the NAT10/ac4C pathway (Figure 5).

### 4.5 Reproductive Endocrine System

NAT10 and ac4C modifications play an important role in oocyte development. NAT10 plays a key role in promoting the timely degradation of polyadenylated mRNAs, including those that encode subunits of the CCR4‐NOT complex, and this activity represents a regulatory step that proves essential for normal oocyte development and successful progression through meiotic prophase I [232]. NAT10 absence in oocytes reduces ac4C on maternal factor mRNAs, causing transcriptomic instability that disrupts the maternal‐to‐zygotic transition [233]. Ac4C modification also plays a regulatory role in spermatogenesis. Specifically ablating NAT10 in germ cells disrupts meiotic prophase I gene expression, impairing meiotic entry and causing defects in synapsis, recombination, and DNA repair [234].

With a broadened scope beyond animal models, investigations into the impact of ac4C modification on the human reproductive process were initiated, utilizing human embryonic stem cells (hESCs). hESCs possess the capacity for unlimited self‐renewal and the capability to differentiate into various cell types representing all three germ layers. These attributes render them a superb in vitro model for investigating mammalian development and disease pathogenesis [235]. It has been found that ac4C modification in the hESC transcriptome is prevalent. Interestingly, the deficiency of NAT10 would significantly perturb hESC self‐renewal and proliferation, while the lack of THUMPD1 and SNORD13 would not [18]. Additionally, it was proposed that NAT10‐mediated ac4C modification could promote ectoderm differentiation of hESCs via acetylating NR2F1 mRNA [236]. Recent studies also developed an independent prognostic model for patient stratification, prognostic evaluation, and prediction of response to immunotherapy in ovarian cancer by classifying OC patients into high‐risk and low‐risk groups [237].

Premature ovarian failure (POF) is characterized by the cessation of ovarian function in women under 40 years of age, clinically presenting as amenorrhea, infertility, reduced estradiol (E2) levels, elevated follicle‐stimulating hormone (FSH), and a depletion of mature follicles [238]. The marked decline in ovarian reserve associated with POF is largely driven by the senescence and cell‐cycle dysregulation of ovarian granulosa cells. Electroacupuncture (EA), a technique combining traditional acupuncture with transcutaneous electrical stimulation, is utilized in clinical practice for its therapeutic potential [239]. Research by Zixiang Geng et al. suggests that EA may alleviate POF by improving the ovarian microenvironment, potentially through downregulating ac4C modification on P16 mRNA. This reduction decreases P16 transcript stability and protein expression, thereby modulating the P16/CDK6/CCND1 signaling axis in granulosa cells [240]. Additionally, RIPK3 expression is elevated in granulosa cells from atretic follicles. In POF models, increased expression of the writer enzyme NAT10 enhances the ac4C‐dependent stabilization of RIPK3 mRNA. This post‐transcriptional regulation influences the Nrf2/HO1 pathway and promotes cellular senescence in ovarian granulosa cells [241].

Endometriosis (EM) conventionally delineates the presence of endometrium‐like tissue beyond the confines of the uterus. Yet, it is acknowledged as a multifaceted inflammatory state influenced by estrogen, unyielding to progesterone. This predicament culminates in dysmenorrhea, infertility, pelvic pain, and reproductive challenges [242]. Certain studies have revealed a noteworthy upregulation of ac4C modification and NAT10 within endometrial lesions in stark contrast to eutopic endometrium, impacting endometrial epithelial cell proliferation, EMT, and cell‐cycle processes. Subsequently, they discerned that ac4C RNA modification bolstered TGFB1 mRNA stability and expression levels. Furthermore, the curbing of NAT10 activity through *Remodelin* significantly halted the proliferation of ectopic lesions in an EM mouse model [243] (Figure 6).

### 4.6 Bone Homeostasis

Bone homeostasis can be simply and accurately defined as the dynamic equilibrium between bone formation and bone resorption [244]. A central aspect of this system is the reciprocal signaling between osteoblasts and osteoclasts, which is regulated by NAT10‐mediated ac4C modification.

Osteoclasts are multinucleated giant cells derived from the myeloid hematopoietic lineage and play an indispensable role in skeletal homeostasis [245]. Their differentiation is accompanied by an increased expression of NAT10, which is further upregulated under inflammatory conditions [246]. However, the functional role of NAT10 in osteoclast differentiation remains controversial. Whereas several studies have reported that the number of TRAP‐positive osteoclasts was unchanged upon NAT10 overexpression or knockdown—thus suggesting that NAT10 does not directly regulate osteoclast differentiation—other studies have convincingly shown that the NAT10 inhibitor *Remodelin* reduces ac4C modification of Fos mRNA and impairs osteoclast differentiation by inhibiting the MAPK signaling pathway [246, 247].

Since osteoblasts have fundamental roles in making collagen‐based matrix and regulating mineralization, it is logical and well‐supported by the literature that NAT10 expression increases during osteogenic differentiation. Both NAT10 expression and ac4C levels are significantly higher during the osteogenic differentiation stage of BMSCs than during the proliferation phase [247]. NAT10 promotes ac4C modification on Gremlin 1 mRNA. Because the protein breaks down more rapidly, there is a drop in Gremlin 1 protein, which therefore relieves its inhibition of BMP/Smad1/5/9 signaling and promotes osteogenic differentiation of MSCs [20]. More importantly, NAT10 directly enhances BMSC osteogenesis by increasing ac4C modification on RUNX2 mRNA, thereby raising the level of this critical osteogenic transcription factor [247]. In hPDLSCs, NAT10 stabilizes VEGFA mRNA via ac4C, elevates VEGFA expression, and activates the PI3K/AKT cascade [248]. Mechanical compression has been clearly shown to increase NAT10 expression, which in turn stabilizes BMP2 mRNA by mediating ac4C modification, thus directly promoting osteogenic commitment of hPDLSCs [249]. From a clinical point of view, this suggests that careful modulation of biomechanical signals could accelerate and improve the quality of bone regeneration. Moreover, the traditional Chinese medicine formula Mijiao has a documented effect on osteogenic pathways involving RUNX2 [250].

The distribution and functional effects of ac4C modification show clear, pathology‐specific patterns in different musculoskeletal disorders. NAT10 is markedly upregulated under inflammatory conditions compared to physiological states, and in LPS‐activated macrophages, NAT10 promotes ROS production by activating the NOX2–ROS–NF‐κB cascade, which in turn potently enhances the secretion of proinflammatory cytokines IL‐6 and TNF‐α [251]. Most importantly, inhibition of NAT10 attenuates osteoclast differentiation and therefore reduces inflammatory bone loss [246].

Rheumatoid arthritis (RA) is a chronic autoimmune disease marked by persistent synovitis and concomitant cartilage and bone destruction, and therefore elevated levels of both NAT10 and ac4C are found in fibroblast‐like synoviocytes (FLSs) and synovial tissues from RA patients [252]. Importantly, NAT10 promotes PTX3 mRNA stability and translational efficiency by means of ac4C modification, and the resulting upregulation of PTX3 strengthens the aggressive phenotype of FLSs [253]. Because heightened immune cell infiltration in synovial tissue accelerates disease progression, it is in sharp contrast to osteoporosis, which is associated with reduced NAT10 expression and lowered global ac4C levels. More importantly, exogenous overexpression of NAT10 has been directly shown to promote bone formation in ovariectomy‐induced osteoporotic models, thus elegantly demonstrating its context‐dependent role in bone metabolism [250].

**FIGURE 7:**
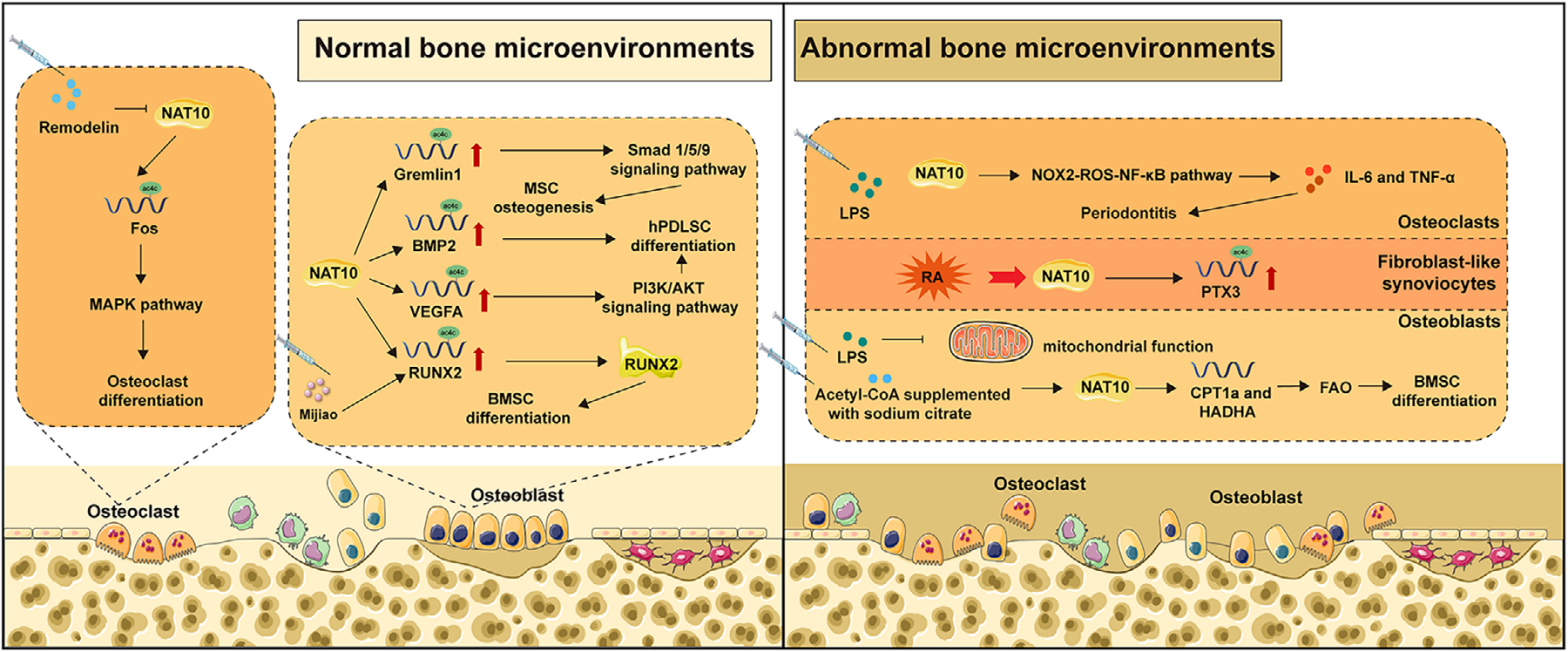
The involvement of ac4C in bone homeostasis. Normal bone microenvironments: Remodeling diminished ac4C modification of Fos mRNA, perturbing the osteoclast differentiation process via the MAPK pathway. NAT10 elevated ac4C levels on Gremlin 1 mRNA to suppress its expression, thereby propelling MSC osteogenesis. NAT10 enhanced VEGFA mRNA stability to activate the PI3K/AKT signaling pathway, fostering hPDLSC osteogenic differentiation. NAT10 mediated compressive force‐induced BMP2 mRNA stabilization via ac4C modification, driving hPDLSC osteogenesis. NAT10 promoted BMSC differentiation by augmenting ac4C levels on RUNX2 mRNA. The traditional Chinese medicine formulation Mijiao also modulated osteogenic differentiation processes mediated by RUNX2. Abnormal bone microenvironments: Stimulation with LPS disrupted mitochondrial function during BMSC osteogenic differentiation. Supplementation of acetyl‐CoA with sodium citrate enhanced NAT10 levels, augmented the mRNA stability and expression of CPT1a and HADHA to boost fatty acid oxidation and rescue impaired osteogenesis of BMSCs under LPS‐induced inflammation. In LPS‐stimulated macrophages, NAT10 activated the NOX2‐ROS‐NF‐κB pathway to heighten the production of inflammatory mediators such as IL‐6 and TNF‐α, ultimately contributing to periodontitis. In RA FLSs, heightened NAT10 enhanced the stability and translation efficacy of N4‐acetylated PTX3 mRNA, further aggravating the injury.

In essence, NAT10 plays a crucial role in maintaining bone homeostasis by regulating ac4C modifications, thereby influencing osteogenesis, osteoclastogenesis, cartilage integrity, and inflammatory bone disorders. Its multifaceted regulatory function mirrors the complexity of the “writer” proteins in cancer biology, positioning it as a central epitranscriptomic regulator in skeletal health and disease (Figure 7).

### 4.7 Other Pathological Diseases

Diabetes is a chronic metabolic disease. It is marked by elevated blood sugar levels, which may result from not making enough insulin or cells not responding adequately to the insulin [254]. Acetylation of tRNA may modulate stress signaling transmission in vivo in mammals. Thumpd1 is critical in the formation of the ac4C at position C12 of tRNA^Ser^ and tRNA^Leu^, but without Thumpd1, the level of tRNA^Leu^ decreases, causing ribosome stalling and eIF2α phosphorylation. Mice missing Thumpd1 showed signs of growth retardation and infertility. Additionally, the combined knockout of Thumpd1 and the stress‐activated kinase Gcn2 caused severe postnatal mortality, suggesting an in vivo interaction between the two genes [255].

Dysregulated adipogenesis is an important trigger of obesity. Research revealed that NAT10 expression was significantly increased in the adipose tissues of obese people and high‐fat‐fed mice. Ac4C modification enhanced the stability of KLF9 mRNA, therefore activating the CEBPA/B‐PPARG pathway to promote adipogenesis [256, 257]. Additionally, ac4C modification of Srebf1 and Scap mRNA modulates hepatic lipogenesis [258]. The root causes of MASLD and MASH predominantly revolve around excessive de novo lipogenesis (DNL) or fatty acid uptake surpassing the capacities for oxidation and very‐low‐density lipoprotein secretion [259]. Sterol regulatory element‐binding protein 1c (SREBP‐1c), a pivotal transcription factor, regulates the majority of rate‐limiting DNL enzymes [260]. The interaction of NAT10 with SREBP‐1c mRNA for acetylation modification enhanced mRNA stability and expression, consequently promoting lipogenic enzymes and fostering MASLD and MASH [261]. *Remodelin* could alleviate MASLD and related metabolic disorders in murine models [261].

Pulmonary fibrosis is a chronic interstitial lung disorder characterized by excessive extracellular matrix accumulation, resulting in tissue scarring and structural disruption [262]. NAT10 could facilitate PM2.5‐triggered lung fibrosis by augmenting ac4C modifications on TGFB1 mRNA, which enhance transcript stability and promote EMT [263].

*Pseudomonas aeruginosa* is a predominant pathogen causing severe pneumonia and acute lung injury (ALI) [264]. Studies demonstrate that NAT10 aggravates *P. aeruginosa*‐induced ALI by mediating ac4C modifications on HMGB1 mRNA, thereby increasing its stability and protein expression. The resulting upregulation of HMGB1 contributes to mitochondrial impairment in lung epithelial cells, ultimately intensifying ALI severity [265].

Inflammatory bowel disease comprises a spectrum of chronic intestinal disorders characterized by persistent inflammation [266]. Investigations have revealed that NAT10 expression was associated with inflammatory and apoptotic cascades in human ulcerative colitis CD4^+^ T cells. The absence of NAT10 led to reduced stability of the antiapoptotic gene BCL2‐associated athanogene 3, triggering a series of responses marked by the elevation of genes linked to apoptosis and an increased rate of apoptosis within T cells [267].

Severe acute pancreatitis represents a profoundly debilitating acute gastrointestinal ailment intricately connected with pyroptosis [268]. Research indicates that NAT10‐mediated ac4C modification also plays a pivotal role in severe pancreatitis. The downregulation of NAT10 results in decreased expression and durability of NOD‐like receptor pyrin domain‐containing protein 3 (NLRP3) mRNA by impeding ac4C modification of NLRP3, consequently suppressing cellular pyroptosis and pancreatic damage in severe acute pancreatitis [269].

In summary, RNA modifications constitute a fundamental regulatory mechanism central to epitranscriptomic governance. This regulatory framework orchestrates a broad spectrum of physiological processes and pathological conditions across diverse biological contexts.

## 5 Therapeutic and Diagnostic Potential

The epitranscriptome constitutes a fundamental regulatory layer that modulates gene expression following transcription. The regulatory network offers druggable targets and clinically relevant biomarkers necessary for disease detection, outcome prediction, and therapy monitoring.

### 5.1 Targeting RNA Modification Machinery for Therapy

Therapeutic strategies targeting RNA modification mechanisms represent one of the core directions in translating epitranscriptomics from basic research to clinical applications. The goal of this field is to correct aberrant gene expression programs in disease states by modulating the writers, erasers, and readers of RNA modifications. When a certain RNA modification activity becomes abnormally active and promotes disease progression, inhibiting its key regulatory proteins is the most direct strategy.

NAT10 is considered an important RNA acetyltransferase and is the only “writer” of the ac4C modification known so far. The use of oxygen therapy is crucial in many situations. NAT10 affects mRNA stability and translation by regulating ac4C modification on RNA, which further affects cell growth, migration, and apoptosis. NAT10 is often regulated in many diseases, including cancers, metabolic disorders, and CVDs. Therefore, specifically targeting NAT10 could be used to modulate these pathological processes.

The inhibition of NAT10 through small‐molecule agents has been investigated using several targeting strategies. In GC models, *Remodelin* lowers MDM2 expression by inhibiting NAT10, which consequently hinders tumor progression [270]. Moreover, combining its inhibition with existing immunotherapies appears promising. Inhibiting NAT10 could potentiate T‐cell function and reduce immunosuppression, potentially yielding synergistic outcomes when used alongside immune checkpoint inhibitors [148].

Nevertheless, clinical translation of NAT10‐targeted therapies faces major hurdles. Genetic variability requires pharmacogenomic‐guided dosing. NAT10 inhibition carries on‐target toxicity risks in healthy tissues, necessitating monitoring. Its pleiotropic effects demand systems‐level studies to define downstream consequences and establish a safe therapeutic window.

A salient gap in the current landscape is the paucity of literature detailing the rational design of therapeutic strategies and specific intervention measures centered on modulating ac4C. This represents not only a specific hurdle for ac4C‐based therapeutics but also a broader challenge constraining the clinical translation of most RNA modifications. Present experimental progress is largely confined to the preclinical tool compound *Remodelin*, with a complete absence of clinical studies validating its safety or efficacy in humans. Future efforts aimed at NAT10 or related epitranscriptomic targets, potentially leveraging the molecular scaffold of inhibitors like *Remodelin*, may guide the development of agents that modulate disease progression through specific influence on the ac4C epitranscriptome.

In summary, therapies that target the epitranscriptome offer a novel strategy for post‐transcriptional regulation, serving as a valuable complement to established genetic and transcriptional treatments. Continued progress in interpreting the epitranscriptomic language and in developing precision‐targeted pharmacological compounds will likely establish this modality as a core element of future precision medicine frameworks.

### 5.2 RNA Modifications as Novel Biomarkers for Early Diagnosis and Prognosis

The dynamic and reversible nature of RNA modifications underscores their regulatory significance in gene expression and disease pathogenesis. Their stability and detectability in accessible biofluids position them as promising noninvasive biomarkers for liquid biopsy, facilitating early diagnosis, risk stratification, and prognostic assessment.

To date, more than 170 chemically distinct RNA modifications have been identified across a broad spectrum of RNA molecules. This extensive chemical repertoire provides a rich resource for identifying disease‐specific markers. The dynamics of these modifications are precisely orchestrated by dedicated writer, eraser, and reader proteins. Notably, the cellular RNA modification profile serves as a sensitive sensor, rapidly reflecting changes in cell state induced by stress, carcinogens, infection, or other pathological triggers. Functionally, RNA modifications exert influence over nearly every stage of the RNA life cycle—including splicing, nuclear export, stability, and translation. Dysregulation of these modifications can lead to the aberrant expression of proto‐oncogenes or tumor suppressor genes, thereby contributing to disease progression [266]. Thus, the detection of specific RNA modifications is intrinsically connected to their biological function. Advanced detection methodologies, such as antibody‐based enrichment techniques (e.g., MeRIP‐Seq) and novel direct sequencing platforms (e.g., ac4C‐seq), now enable comprehensive, genome‐wide mapping of modification landscapes [42, 271]. Given the distinct characteristics of RNA modifications and continuous technological advances, they represent a highly promising class of biomarkers for early diagnostic and prognostic applications.

RNA modifications have considerable potential as clinical biomarkers. Future work must adopt rigorous validation protocols and address key methodological and biological constraints.

## 6 Challenges, Open Questions, and Future Directions

Although numerous studies have been conducted on RNA modifications, several issues and challenges remain to be addressed.

### 6.1 Technical Hurdles in Precise Mapping and Single‐Cell Analysis

Precise mapping typically refers to whole‐transcriptome, single‐base‐resolution modification detection. Accurate mapping and single‐cell analyses are critical technological frontiers moving RNA modification research from “population averages” toward “cellular heterogeneity” and “absolute precision,” but they face severe technical challenges.

Current approaches for mapping RNA modifications with precision predominantly rely on immunoenrichment‐based techniques. Mainstream methods, including MeRIP‐Seq, utilize modification‐specific antibodies for affinity purification. The accuracy of these techniques is inherently limited by the antibody's enrichment efficiency, specificity, and sequence bias, which collectively introduce artifacts and generally prevent mapping at single‐nucleotide resolution. Additionally, as population‐level assays, they cannot determine allelic specificity—whether a modification is present on one or both alleles at a heterozygous site. Their output is typically a relative measure of enrichment rather than an absolute quantification of the modified molecule's abundance. Therefore, a fundamental quantitative question in epitranscriptomics remains unresolved: determining the precise fraction of transcripts that are modified at a given genomic locus. This represents a significant methodological gap in achieving quantitative, allele‐resolved epitranscriptomic mapping.

Another limitation of these methods is that nearly all second‐generation sequencing‐based methods rely on PCR amplification. PCR amplification distorts the true abundance of RNA modifications and can even completely erase the signal for some modifications. In addition, most current methods are limited to detecting one modification per experiment. The need for separate assays to map multiple modifications comprehensively consumes large amounts of biological material and poses significant challenges for data integration. To overcome these technical challenges, antibody‐independent, chemistry‐based detection strategies—such as ac4C‐seq—could be developed. The principle here is to use specific chemical “click” reactions with modified bases to generate mutations or termination during reverse transcription. These strategies can be used upstream of high‐throughput sequencing to detect these signals. In addition, more effective bioinformatics tools could be developed for the accurate identification of modification sites from chemical‐based data. They could also be used to calculate modification rates.

The main goal of single‐cell RNA modification analysis is to study RNA modifications at the individual cell level. In other words, it reveals cellular heterogeneity. For example, differences in the tumor microenvironment or among complex cell types in the brain. Nonetheless, there are still several technical hurdles to clear. To begin with, the extremely low starting sample size found in each individual cell makes direct detection challenging, while scaling down techniques that work on bulk cells (such as antibody enrichment) to the single‐cell level is highly challenging. During enrichment, many steps involve washing and purifying the sample. This leads to considerable loss of RNA, subsequently making the analysis impossible. Moreover, the simultaneous loss of transcriptomic and epitranscriptomic data excludes crucial context for gene expression following sequencing. A principal technical and bioinformatic challenge is carrying out RNA modification analysis on thousands of individual cells efficiently and incorporating these data with gene expression and even surface protein markers.

### 6.2 The Complexity of the “Modification Code”: Crosstalk and Context‐Dependency

The human organism constitutes a highly integrated system characterized by extensive communication across its constituent tissues and organs. This systemic complexity is mirrored at the molecular level by the epitranscriptome. Different modification types, or even distinct modification sites on the same RNA transcript, engage in mutual regulation and crosstalk.

For example, there is a possibility that ac4C is connected to other RNA modifications. According to this notion, NAT10 mediates ac4C modification on YTHDC1 mRNA, which enhances YTHDC1 expression and stabilizes its mRNA. YTHDC1 recognizes two m^6^A locations within mRNAs for the enzymes PFKM and LDHA. The recognition by tumor cells increases the stability of these mRNAs in an m^6^A methylation‐dependent manner, as well as glycolytic disassembly [125]. This is an unexplored area that could become a research focus. Additionally, there has not been much convenient and rapid development of molecular biological detection approaches, except for ac4C‐seq, to identify ac4C acetylation sites.

Moreover, the biological role of a given RNA modification at a defined site is not predetermined but is strongly influenced by its molecular and cellular context. The functional consequence of a modification can vary markedly depending on its position within a transcript, exemplifying the context‐dependent nature of epitranscriptomic regulation.

While the precise mechanism through which ac4C influences mRNA turnover is yet to be fully elucidated, it is noteworthy that m6A marks within the 3′ UTR of MYB and MYC transcripts can accelerate their decay, thereby promoting leukemogenesis [272]. This observation supports a model in which NAT10‐dependent ac4C deposition may similarly modulate mRNA stability, potentially through interactions involving 3′ UTR regulatory elements.

NAT10 acetyltransferase was the first to be associated with ac4C modification [16]. Earlier studies showed contrasting effects of NAT10 on the same signaling pathway. One study showed that NAT10 can acetylate MDM2 mRNA, thus reducing levels of p53 protein and increasing levels of MDM2 protein. On the other hand, another report revealed that NAT10 acetylated p53, preventing ubiquitination by MDM2 and increasing the stability of p53 [270, 273]. These results show the dependence of NAT10's cellular functions on context. The fact that NAT10 provides ac4C modification of RNA in a tissue‐specific fashion is consistent with effects that differ between cell types and genes within the same cell. An experiment with 293T cells demonstrated that NAT10 catalyzed the ac4C modification of HIV transcripts, improving mRNA stability [82]. Conversely, in MSC cell studies, the enhancement of the ac4C modification by NAT10 reduced the stability of Gremlin 1 mRNA [20].

Furthermore, the functional consequence of an m6A modification is determined by which YTH domain‐containing protein (such as YTHDF1, YTHDF2, YTHDF3, or the nuclear‐localized YTHDC1) binds at the site. The recruitment and functional output of these reader proteins are further regulated by factors that include their subcellular distribution, relative abundance, and interactions with auxiliary proteins and signaling pathways.

In summary, interpreting epitranscriptomic regulation demands an integrated analytical perspective, given that biological outcomes emerge from complex interplay and are highly sensitive to cellular context.

### 6.3 From Association to Causality: Unraveling Precise Molecular Mechanisms

A typical framework begins with identifying associations between modifications and biological states, validated by controlled experiments and sequencing. At the cellular level, phenotypes are linked to candidate genes via perturbation. A key step is confirming pathway dependence: writer or eraser enzymes modify specific target mRNAs, leading to altered expression of downstream genes. Once causality is established, mechanistic studies examine effects on RNA half‐life, trafficking, or translation. Final validation in animal models or clinical cohorts confirms biological relevance and translational potential.

However, current research still has many unresolved issues. Taking ac4C modification as an example, even though ac4C acetylation has attracted significant attention, many gaps remain. Many studies have explored the role of ac4C in transcription and translation and how it affects phenotypes in disease conditions, but less is known about its function in normal physiological settings. Moreover, disease models are mostly restricted to cancer and no other conditions. Researchers who want to conduct experiments must have expertise and refined skills. Similarly, data analysis requires professionals. Moreover, there is no widely accepted and effective computational detection method in this field. The result of continuous investigation of RNA modification is anticipated to bring more understanding and elucidate the mechanism and important functions of ac4C in the near future.

## 7 Conclusion

The regulation of gene expression through RNA modifications parallels classical epigenetic mechanisms exemplified by histone acetylation, a well‐known hallmark of active chromatin states [274]. Across diverse organisms—including bacteria, eukaryotic cells, and certain viruses—the ac4C RNA modification shows a dual, context‐sensitive nature, characterized by variable tissue distribution and effects that shift between advantageous and harmful depending on physiological or disease contexts. This acetylation moiety critically modulates RNA structural stability and translational yield, processes integral to protein synthesis and metabolic regulation. Taking ac4C as a focal model, this article systematically surveys its enzymatic modifiers, detection methodologies, and varied physiological contributions.

Epitranscriptomics has unveiled a dynamic, multilayered regulatory architecture that operates beyond conventional transcription‐based control. This field is transitioning from early efforts in modification mapping toward elucidating underlying regulatory networks and functional relevance in living systems. Future studies will need to address the temporal regulation of RNA marks, their interactive signaling with other cellular pathways, and their specific functions within distinct biological settings.

Several limitations of the present review should be noted. First, the rapidly advancing pace of ac4C research means that not all recent findings may be incorporated, and the synthesis may reflect selective emphasis within the published literature. Second, interpretations offered here are constrained by the methodological designs and technical capabilities of the primary studies referenced. Lastly, while detection tools for ac4C continue to improve, gaps persist in our mechanistic understanding, indicating the necessity of further empirical and theoretical work to refine current models.

## Author Contributions

Yueqi Chen, Zhao Xie, Fei Luo, and Shiyu Xiao designed the manuscript; Shiyu Xiao, Tingwen Xiang, and Chuan Yang wrote the original draft; data collection, analysis, and interpretation by Shiyu Xiao, Tingwen Xiang, Chuan Yang, Xiaohua Wang, and Gang Huang. Shiyu Xiao, Tingwen Xiang, Chuan Yang, Fei Luo, Zhao Xie, and Yueqi Chen revised the draft. All the authors approved the final version of the manuscript.

## Funding

This work was supported by National Natural Science Foundation Regional Innovation and Development Joint Fund (No. U23A20413), National Natural Science Foundation of China (No. 82471836, 82202707), Key Clinical Cultivation Discipline Construction Project (No. 145AHQ141009000X), Young Elite Scientist Sponsorship Program by Association for Science and Technology of Qinghai Province (No. 2024QHSKXRCTJ35), and Natural Science Foundation of Chongqing (No. CSTB2025NSCQ‐GPX0572).

## Ethics Statement

The authors have nothing to report.

## Conflicts of Interest

The authors declare no conflicts of interest.

## Data Availability

All data are available from the corresponding authors upon reasonable request.
